# Nuclear respiratory factor 1 drives hepatocellular carcinoma progression by activating LPCAT1-ERK1/2-CREB axis

**DOI:** 10.1186/s13062-023-00428-z

**Published:** 2023-10-24

**Authors:** Ran Liu, Chuanzheng Yin, Peng Zhao, Bing Guo, Wenbo Ke, Xichuan Zheng, Dawei Xie, Yaofeng Wang, Gengqiao Wang, Yinzhao Jia, Yang Gao, Wenjun Hu, Gang Logan Liu, Zifang Song

**Affiliations:** 1 Department of Hepatobiliary Surgery, Union Hospital, Tongji Medical College, Huazhong University of Science and Technology, 1277 Jiefang Avenue, Wuhan, 430022 Hubei China; 2 Insitute for Genome Sciences, University of Maryland School of Medical, Baltimore, MD 21201 USA; 3 State Key Laboratory of Molecular Oncology, National Cancer Center/National Clinical Research Center for Cancer/Cancer Hospital, Chinese Academy of Medical Sciences and Peking Union Medical College, Beijing, 100021 China; 4 School of Life Science and Technology, Huazhong University of Science and Technology, Wuhan, 430074 Hubei China

**Keywords:** NRF1, LPCAT1, Hepatocellular carcinoma, Progression, Positive feedback loop

## Abstract

**Background:**

Nuclear respiratory factor 1 (NRF1) is a transcription factor that participates in several kinds of tumor, but its role in hepatocellular carcinoma (HCC) remains elusive. This study aims to explore the role of NRF1 in HCC progression and investigate the underlying mechanisms.

**Results:**

NRF1 was overexpressed and hyperactive in HCC tissue and cell lines and high expression of NRF1 indicated unfavorable prognosis of HCC patients. NRF1 promoted proliferation, migration and invasion of HCC cells both in vitro and in vivo. Mechanistically, NRF1 activated ERK1/2-CREB signaling pathway by transactivating lysophosphatidylcholine acyltransferase 1 (LPCAT1), thus promoting cell cycle progression and epithelial mesenchymal transition (EMT) of HCC cells. Meanwhile, LPCAT1 upregulated the expression of NRF1 by activating ERK1/2-CREB signaling pathway, forming a positive feedback loop.

**Conclusions:**

NRF1 is overexpressed in HCC and promotes HCC progression by activating LPCAT1-ERK1/2-CREB axis. NRF1 is a promising therapeutic target for HCC patients.

**Supplementary Information:**

The online version contains supplementary material available at 10.1186/s13062-023-00428-z.

## Background

Hepatocellular carcinoma (HCC) is the second most lethal cancer worldwide.[1] Despite progress in tumor treatment, the prognosis of HCC remains to be poor mainly because most of HCC cases are diagnosed at late stage. Since surgery was not applicable for majority of HCC patients, [2] it’s urgent to find novel therapeutic targets to improve treatment efficacy.

Nuclear respiratory factor 1 (NRF1) is a transcription factor that controls the biogenesis of mitochondria. It dimerizes and binds to palindromic promoter sites to activate the transcription of many genes indispensable for mitochondria, such as mitochondrial transcription factor A (TFAM), cytochrome c. [3] As a central metabolic organ, liver is rich in mitochondria. Consistently, by regulating mitochondrial dynamics, NRF1 participates in several liver diseases. NRF1-TFAM axis regulates mitochondrial biogenesis and protects against mitochondrial dysfunction in alcoholic steatohepatitis.[4] NRF1 expression and NRF1-mediated mitochondrial biogenesis in liver cells are both upregulated in acute inflammation to meet the energy demand.[5] However, little is known about its function in HCC. Besides regulating mitochondrial biogenesis, NRF1 was also demonstrated to control the expression of genes that are associated with DNA replication, cell proliferation and apoptosis.[6] Accumulating evidence showed NRF1 might participate in tumor progression. For instance, in breast cancer, NRF1 was reported to promote spheroid survival and mesenchymal transition.[7] NRF1 also activates C-X-C motif chemokine receptor 4 (CXCR4) transcription and is responsive to estrogen in breast cancer.[8, 9] Moreover, bioinformatic analysis implicated that NRF1 is highly active in glioblastoma and NRF1 promotes the proliferation of renal cancer cells by directly regulating transcription factor binding to IGHM enhancer 3 (TFE3).[10, 11] In contrast to these findings, NRF1 was also reported to act as a tumor suppressor. It suppresses transforming growth factor β (TGF-β) signaling and inhibits the proliferation and migration of PC3 prostate cancer cells.[12] NRF1 also regulates the expression of autophagy related 5 (ATG5) and autophagy related 7 (ATG7) and inhibits the migration of melanoma cells.[13] Thus, NRF1 is not only a mitochondrial biogenesis stimulator but also a transcription factor that is involved in multiple cellular activities. Nevertheless, its role in HCC and the comprehensive regulatory mechanisms are elusive and need to be investigated.

Lysophosphatidylcholine acyltransferase 1 (LPCAT1) is a kind of enzyme that reshapes cell membrane phosphatidylcholine (PC). After PC are de novo synthesized, their fatty acyl chains at the sn-2 site are hydrolyzed to produce lysophosphatidylcholine (LPC) which are then reacylated by LPCATs to incorporate another fatty acid to the sn-2 position and form a new PC species [14]. LPCAT family contains four members (LPCAT1-LPCAT4) and they have different tissue distributions, enzymatic activities, and substrate preferences.[14] Among them, LPCAT1 prefers 16:0-CoA (palmitoyl-CoA) as its substrate and its main catalytic product is dipalmitoyl phosphatidylcholine (DPPC) which facilitate signal transduction from membrane to cytosol.[15] LPCAT1 was reported to promote HCC progression,[16] but how it’s upregulated in HCC remains unclear.

In the present study, we found that NRF1 is aberrantly overexpressed and overactive in HCC. Knockdown of NRF1 inhibited the proliferation and migration of HCC cells and vice versa. Mechanistic study showed that NRF1 activated ERK1/2-CREB signaling pathway by promoting LPCAT1 transcription and LPCAT1 reciprocally promoted NRF1 expression through ERK1/2-CREB signaling pathway via its catalysate DPPC. Therefore, NRF1 is a potential therapeutic target of HCC.

## Materials and methods

### Bioinformatic analyses

HCC transcriptome sequencing data and clinical information were downloaded from TCGA (https://portal.gdc.cancer.gov/). There were 424 samples in total, with 49 samples of normal tissue and 373 samples of tumor tissue. The data were then analysed and visualized in R software. Gene sets from MSigDB and GSEA software (http://www.gsea-msigdb.org/gsea/index.jsp) were used to conduct GSEA. The parameters in GSEA were set as default: number of permutations = 1000, permutation type = phenotype, max size = 500, min size = 15. NRF1 target genes inferred from ChIP-seq data were downloaded from ChIP-Atlas (http://chip-atlas.org/) which integrated 22 NRF1 ChIP-seq experiments in various cell lines and target genes were ranked by the average coverage within 1000 bp from TSS. The most differential survival genes which displayed the smallest *p* value in survival analysis in HCC were downloaded from GEPIA2 (http://gepia2.cancer-pku.cn/#survival).

### Patients and tissue specimens

Human HCC tumor and adjacent non-tumor tissue (*n* = 65) were collected from HCC patients who underwent hepatectomy at the Department of Hepatobiliary Surgery, Union Hospital affiliated to Tongji medical college, Huazhong University of Science and Technology (Wuhan, China) from October 2020 to April 2022. All procedures were approved by the Ethics Committee of Union Hospital, and followed the Declaration of Helsinki Principles. Written informed consent was obtained from all patients prior to surgery. Each sample were incised into two pieces and one was dipped in formalin for tissue microarray and the other was kept in liquid nitrogen for protein or RNA extraction.

### Tissue microarray and immunohistochemistry

The human HCC or adjacent normal liver tissue were embedded in paraffin and a piece of each sample was taken and put together to make a tissue microarray for further analysis. The human HCC tissue microarray was deparaffinized with xylene and rehydrated with sequential gradients of ethanol. The antigen-retrieved and blocked section was incubated with NRF1 (A3252, 1:2000 dilution, ABclonal) antibody. Cross-sections of subcutaneous tumors from nude mice were incubated with primary antibodies against NRF1 (A3252, 1:2000 dilution, ABclonal), LPCAT1 (A4987,1:2000 dilution, ABclonal), p-ERK1/2 (Thr202/Tyr204) (4370, 1:50 dilution, Cell Signaling Technology), p-CREB (Ser133) (9198, 1:50 dilution, Cell Signaling Technology), Ki67(27,309–1-AP, 1:3000 dilution, Proteintech) and Vimentin (5741, 1:1000 dilution, Cell Signaling Technology). Subsequently, the sections were incubated with HRP conjugated secondary antibodies and stained with 3, 3′- diaminobenzidine (DAB), with hematoxylin counterstaining the nucleus. Two trained pathologists were blinded to evaluate the immunostaining. The IHC scores were assigned as the product of intensity (0, negative; 1, weak; 2, moderate; 3, strong) and frequency of positive cells (0, less than 5%; 1, 5%-25%; 2, 26%-50%; 3, 51%-75%; 4, more than 75%) with a range of 0–12.

### Cell culture and chemical treatment

Human HCC cell lines HepG2, Huh7, SNU182 were purchased from Procell (Wuhan, China), human HCC cell line MHCC97H and immortalized normal human liver cell line MIHA were purchased from FENGHUISHENGWU (Changsha, China). All the cell lines had STR certification reports. The cells were all maintained in high glucose DMEM (Procell, Wuhan, China), supplemented with 10% fetal bovine serum (Life Technology, Grand Island, USA) and 100U/mL penicillin–streptomycin (Invitrogen, Carlsbad, USA) at 37 °C and 5% CO2.

For the inhibition of PI3K, NF-κB, ERK1/2, cells were treated with LY294002 (HY-10108, 20 μM, MedChemExpress), Bay 11–7082 (HY-13453, 10 μM, MedChemExpress) and PD184352 (HY-50295, 5 μM, MedChemExpress), respectively.

For the supplementation of exogenous DPPC, cells were treated with DPPC (HY-109506, 10 μM, MedChemExpress).

### Cell transfection and lentivirus infection

Plasmids containing shRNAs targeting NRF1 or LPCAT1, plasmids encoding full length human NRF1 or LPCAT1, luciferase plasmids with LPCAT1 promoter were purchased from GenePharma (Suzhou, China), lentivirus packaging plasmids psPAX2 and pMD2G were purchased from Tsingke (Wuhan, China). The specific target plasmids were cotransfected with lentivirus packaging plasmids into HEK293T cells using lipo8000 (Beyotime, Shanghai, China). The medium was replaced with fresh medium 24 h after transfection and the supernatant containing lentivirus was collected 48 h after transfection. After being filtered through 0.22 μm filter, the medium was added to Huh7 and MHCC97H and selected for stable cells with puromycin (Beyotime, Shanghai, China). For CREB knockdown, a siRNA was purchased from GenePharma (Suzhou, China) and transfected into cells with lipo8000. The sequences of the shRNAs and siRNAs are listed in Additional file 9: (Table S1).

### qRT-PCR

Total RNA was extracted using TRIzol reagent (Invitrogen, California, USA). Reverse transcription was conducted using PrimeScript RT Master Mix (Takara, Shiga, Japan) and an iCycler Real-Time PCR Detection System (Bio-Rad, California, USA) were utilized to perform quantitative reverse-transcriptase (RT)-PCR with SYBR Premix Ex Taq kit (Takara, Shiga, Japan). The mRNA levels were normalized by β-actin and calculated with the 2-ΔΔCt method. The sequences of primers were listed in Additional file 10: (Table S2).

### Western blot

Proteins were extracted with RIPA lysis buffer (Beyotime, Shanghai, China) followed by sonication. The proteins were separated with SDS-PAGE electrophoresis and transferred from gel onto PVDF membranes. After being blocked by skim milk, PVDF membranes were incubated with corresponding primary antibodies against NRF1 (A5547, 1:2000 dilution, ABclonal), LPCAT1 (A4987,1:2000 dilution, ABclonal), β-actin (66,009–1-Ig, 1:5000 dilution, Proteintech), Cyclin D1 (ab40754, 1:1000 dilution, Abcam), CDK4 (ab7955, 1:1000 dilution, Abcam), Cyclin E1 (ab71535, 1:2000 dilution, Abcam), CDK2 (ab32147, 1:1000 dilution, Abcam), E-cadherin (3195, 1:1000 dilution, Cell Signaling Technology), N-cadherin (13,116, 1:1000 dilution Cell Signaling Technology), Vimentin (5741, 1:1000 dilution, Cell Signaling Technology), p-ERK1/2 (Thr202/Tyr204) (4370, 1:1000 dilution, Cell Signaling Technology), ERK1/2 (4695, 1:1000 dilution, Cell Signaling Technology), p-CREB (Ser133) (9198, 1:1000 dilution, Cell Signaling Technology), CREB (9197, 1:1000 dilution, Cell Signaling Technology), PCNA(13,110, 1:1000 dilution, Cell Signaling Technology). Then the membranes were incubated with corresponding HRP-conjugated AffiniPure Goat Anti-rabbit (mouse) IgG (BA1055, 1:5000 dilution, Boster Biological Technology). The proteins were finally visualized and recorded by the ChemiDoc imaging system (Bio-Rad, California, USA). β-actin served as the loading control.

### Mitochondrial function analysis

For glucose uptake and lactate secretion analysis, cells were seeded into 6-well plates (50,000 cells per well). After attachment, the medium was changed to fresh medium. After incubation for 6 h, the medium was collected. Glucose concentration was measured by Hitachi 7180 Chemistry Analyzer. Lactate concentration was measured by VITROS 5600 Integrated System. The glucose or lactate concentration at 0 h or 6 h was used to calculate glucose uptake and lactate secretion rate.

For ATP level and NAD + /NADH ratio analysis, cells were seeded into 6-well plates (50,000 cells per well). After attachment, ATP level detection kit (Beyotime, S0026) and NAD + /NADH ratio detection kit (Beyotime, S0175) was used according to manufacturer's instructions.

### CCK8 assay

The cells were seeded into 96-well plates (2000 cells with 100 μl DMEM per well). The cells were cultured for indicated time and the medium was replaced with 100 μl DMEM containing 10 μl CCK8 solution (Beyotime, Shanghai, China) and incubated in dark for 30 min. The absorbance at 450 nm was measured with Multiskan™ GO microplate spectrophotometer (Thermo Fisher Scientific, Gillingham, UK).

### Colony formation assay

The cells were seeded in 6-well plate (1000 cells with 2 ml complete medium per well) and cultured for 10 days. Then the cells were fixed with 4% paraformaldehyde (30 min at room temperature) and stained with crystal violet (30 min at room temperature).

### Flow cytometry

For cell cycle distribution, cells were collected and washed twice with PBS and then fixed with 70% ethanol overnight and stained with propidium iodide (PI) for 30 min before analysis. For apoptosis analysis, cells were collected with EDTA-free trypsin and washed twice with PBS and stained with PI and Annexin V-APC. The samples were analysed using a FACS flow cytometry (Becton, Dickinson and Company, New Jersey, USA). The data were processed with FlowJo software.

### Transwell migration and invasion assay

For migration assay, we used 8 μm pore 24-well Corning Costar inserts. For invasion assay, the insert was coated by matrigel prior to use. 3 cancer cells into the upper chamber with 200 μl FBS-free DMEM. The lower chamber contained 600 μl 20%-FBS DMEM. The cells were cultured for indicated time (24 h for migration and 48 h for invasion) and fixed with 4% paraformaldehyde (30 min at room temperature) and stained with crystal violet (30 min at room temperature). Then four random sights per well were photographed using a microscope. The images were processed by FIJI software for cell counting.

### Wound healing assay

Cells were seeded into 6-well plates (1 × 10^6^ cells with 2 ml complete medium per well) and cultured for 24 h to let cells grow to 100% confluence. Then the cell monolayer was scratched with a 100 μl pipette tip to make wounds. The wounds were photographed at indicated time points with a microscope and the image were processed with FIJI software.

### ChIP-qPCR

Chromatin immunoprecipitation (ChIP) was performed with SimpleChIP® Plus Sonication Chromatin IP Kit (#56,383, Cell Signaling Technology) according to the manufacturer’s instructions. The NRF1 (A3252, ABclonal) and CREB (9197, Cell Signaling Technology) antibody were used. The purified DNA was analysed by quantitative PCR with SYBR Premix Ex Taq kit (Takara, Shiga, Japan). The primers were listed in Additional file 11: (Table S3).

### Dual luciferase reporter assay

Firefly luciferase plasmid with LPCAT1 wild-type promoter and control Renilla luciferase plasmid were transfected into Huh7 and MHCC97H cells which were previously infected with lentivirus to silence or overexpress NRF1. After 48 h, the cells were harvested and luciferase activity were measured with a dual-Luciferase reporter assay system.

### Nude mice xenograft tumor model

For tumor growth assessment, Huh7 cells (5 × 10^6^) that were infected with NRF1 knockdown lentivirus and control virus were inoculated subcutaneously into the right flank of nude mice (BALB/c nu, five weeks, male, n = 6 per group). The tumor volume was measured every three days (V = 0.5 × L × W^2^, where L is length and W is width). 21 days later the mice were sacrificed and the tumors were excised and weighed. The tumors were then subjected to Western blot and IHC analysis to detect relevant protein expression.

For tumor metastasis assessment, MHCC97H cells (1 × 10^6^ in 200 μl PBS) that were infected with NRF1 knockdown lentivirus and control virus were injected through lateral tail vein of nude mice (BALB/c nu, five weeks, male, n = 6 per group). Six weeks later, the mice were sacrificed and the lungs were excised. Photographs and HE staining were utilized to evaluate tumor metastasis ability.

### Statistical analysis

All experiments were performed at least for three times. Data were shown as mean ± standard difference (SD). Firstly, Shapiro–Wilk normality test was applied. For nonnormally distributed data, Mann–Whitney test was used. For normally distributed data, if the data had equal variance, two tail unpaired student’s t test was used; if not, two tail unpaired t test with Welch’s correction was used. One way ANOVA was applied for more than 2 groups. Survival was assessed via Kaplan–Meier approach with a log-rank test. The analyses were performed in GraphPad Prism 9 software and p < 0.05 was considered statistically significant.

## Results

### NRF1 is highly upregulated in HCC and correlated with poor prognosis of patients with HCC

**Fig. 1:**
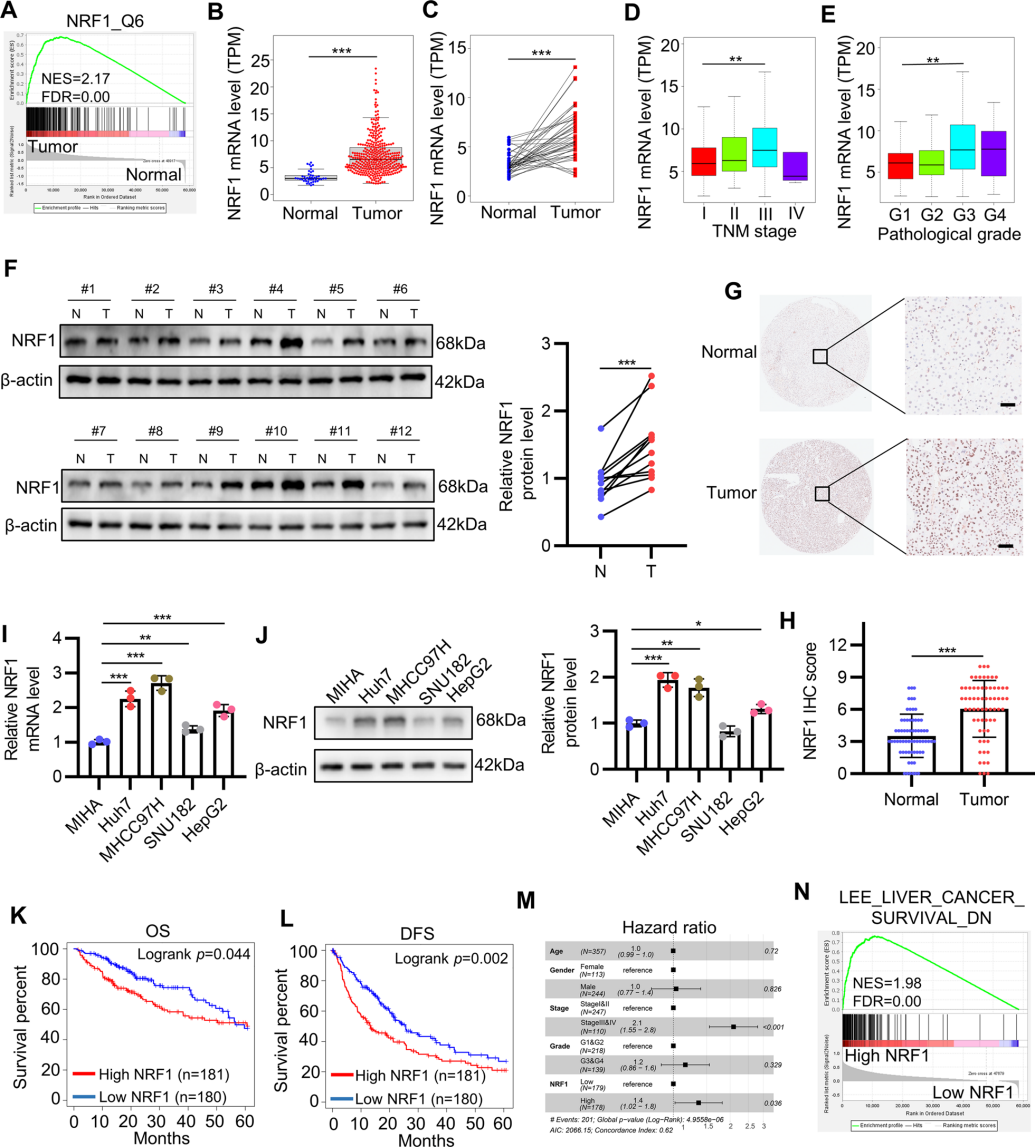
NRF1 is highly upregulated in HCC and correlated with poor prognosis of patients with HCC. **A** GSEA was applied to compare NRF1 activity in HCC and normal tissue in TCGA-LIHC dataset. **B**-**C** NRF1 mRNA level was compared between tumors and normal tissue (**B**) or paired tumors and adjacent normal tissue (**C**) in TCGA-LIHC dataset. **D**-**E** The correlation of NRF1 mRNA level and TNM stage (**D**) or pathological grade (**E**) in TCGA-LIHC dataset. **F** NRF1 expression in our HCC tissue and adjacent normal tissue was determined by Western blot. **G**-**H** The representative IHC images of NRF1 was exhibited (**G**) and the IHC score (*n* = 65) was shown (**H**). Scale bar: 50 μm. **I**-**J** NRF1 mRNA and protein levels in HCC cell lines and normal liver cell line were analysed by qRT-PCR (**I**) and Western blot (**J**). **K**-**L** Kaplan–meier curve showed the effect of NRF1 on HCC overall survival (K) and disease free survival (**L**) in TCGA-LIHC dataset. **M** Forest plot showed Cox proportional hazards model for disease free survival in TCGA-LIHC dataset. **N** The relevance of NRF1 expression and HCC patients survival was analyzed with GSEA in TCGA-LIHC dataset. *p < 0.05, **p < 0.01, ***p < 0.001

We firstly utilized transcriptome sequencing data from TCGA-LIHC cohort to screen hyperactive transcription factors in HCC. By performing gene set enrichment analysis (GSEA) with transcription factor targets gene sets, we found NRF1_Q6 gene set (all the genes whose promoter region contains NRF1 binding motif) was significantly enriched in tumor group compared with normal tissue group (Fig. 1A), which demonstrated that the majority of NRF1 target genes were significantly overexpressed in HCC tumor tissue, indicating NRF1 was functionally hyperactive in tumor tissue compared with normal hepatic tissue. We then focused on NRF1 and performed further bioinformatic analyses based on TCGA-LIHC data to explore its role in HCC. We found NRF1 mRNA level was significantly elevated in tumor tissue compared with normal tissue (Fig. 1B), which was the same for the paired HCC tissue and adjacent normal tissue (Fig. 1C). Furthermore, NRF1 mRNA level was gradually elevated in tumor tissue as the TNM stage or pathological grade increased (Fig. 1D and 1E). Then, we sought to verify the upregulation of NRF1 in our HCC tissue specimen and cell lines. As expected, Western blot and immunohistochemistry (IHC) results showed that NRF1 protein expression was significantly higher in HCC tumor tissue than in adjacent normal tissue (Figs. 1F-H). Similarly, both mRNA and protein level of NRF1 were elevated in four HCC cell lines (HepG2, Huh7, MHCC97H, SNU182) compared with the immortal normal liver cell line (MIHA) (Fig 1 I and J). We wondered whether NRF1 upregulation correlated with prognosis of HCC patients. Predictably, survival analysis using TCGA-LIHC data indicated that patients of high NRF1 expression had shorter overall and disease free survival (Fig. 1K and L), which was further supported by cox proportional hazard model for disease free survival (Fig. 1M). Likewise, GSEA result also showed that genes indicating poor survival in HCC were enriched in high NRF1 group, implying that higher NRF1 changed the gene expression pattern of HCC to a more aggressive phenotype (Fig. 1N). Taken together, these results suggest that NRF1 is highly upregulated in HCC and correlated with poor prognosis of patients with HCC.

### NRF1 promotes HCC cell growth by inducing G1-S cell cycle transition

**Fig. 2:**
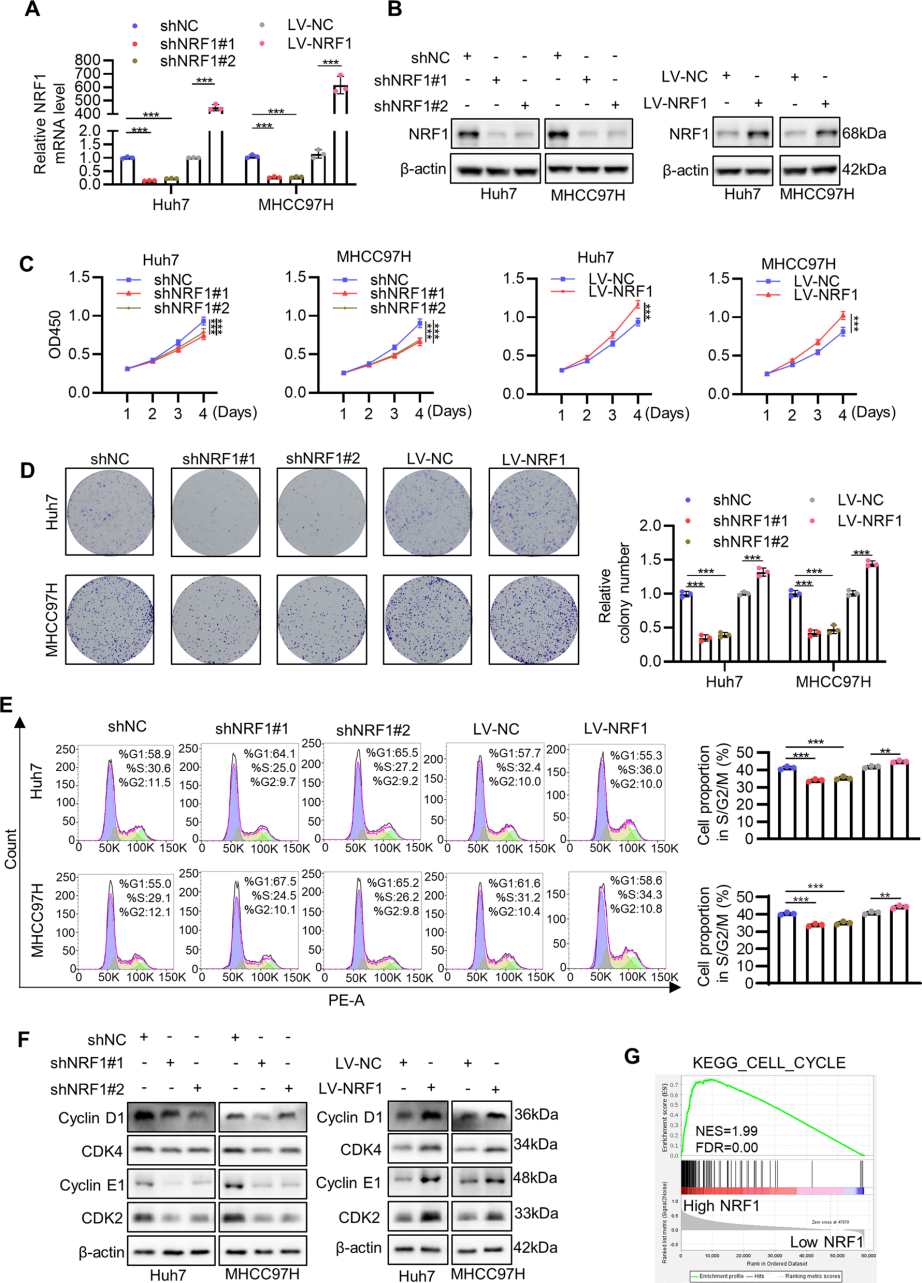
NRF1 promotes HCC cell growth by inducing G1-S cell cycle transition. **A**-**B** NRF1 knockdown (shNRF1#1 and shNRF1#2; shNC as negative control) and overexpression (LV-NRF1; LV-NC as negative control) efficiency were determined by qRT-PCR (A) and Western blot (**B**). **C** Cell proliferation of Huh7 and MHCC97H after NRF1 knockdown or overexpression was detected by CCK8 assay. **D** Colony formation assay was conducted with Huh7 and MHCC97H after NRF1 knockdown or overexpression. The bar graph showed the quantitative results. **E** Cell cycle distribution was analysed by flow cytometry with propidium iodide staining. The relative cell cycle proportion was shown in the bar graph. **F** Cell cycle driver genes protein level were analysed by Western blot after NRF1 knockdown or overexpression. **G** GSEA using KEGG cell cycle gene set between high NRF1 and low NRF1 group in TCGA-LIHC dataset. *p < 0.05, **p < 0.01, ***p < 0.001

Based on the finding that NRF1 was upregulated in HCC, we determined to characterize its biological function in HCC cells. We selected Huh7 and MHCC97H cell lines to perform the following experiment because they have relatively high expression of NRF1. We first utilized lentivirus to knock down NRF1 (shNRF1#1 and shNRF1#2; shNC as negative control) or overexpress NRF1 (LV-NRF1; LV-NC as negative control) in the two cell lines. After establishing stable cell lines, we utilized qRT-PCR (Fig. 2A) and Western blot (Fig. 2B and S1A) to verify the knockdown and overexpression efficiency. As NRF1 plays a critical role in mitochondrial biogenesis, we also validated mitochondrial function. ATP level, glucose uptake rate and NAD + /NADH ratio decreased while lactate secretion rate increased upon NRF1 knockdown (Figures S1B-S1E), demonstrating impaired oxidative phosphorylation and compensatory upregulated glycolysis. Next, we used CCK8 assay to evaluate cell proliferation and the result showed that knockdown of NRF1 significantly inhibited Huh7 and MHCC97H proliferation, and overexpression of NRF1 had the opposite effects (Fig. 2C). Colony formation assay further revealed that knockdown of NRF1 decreased the colony formation rate and vice versa (Fig. 2D). We then conducted flow cytometry analysis to evaluate cell cycle distribution. As shown in Fig. 2E, knockdown of NRF1 decreased, while overexpression of NRF1 increased the cell proportion in S/G2/M phase. Consistently, Western blot results indicated that NRF1 knockdown downregulated the expression of fundamental cell cycle drivers including cyclin D1, cyclin E1, CDK2, CDK4, which were upregulated upon NRF1 overexpression (Figs. 2F and S2A-S2D). These results were further supported by GSEA result of TCGA-LIHC cohort, in which cell cycle-related genes were significantly enriched in high NRF1 group (Fig. 2G). Moreover, flow cytometry also indicated that NRF1 did not influence cell apoptosis significantly (Figures S2E and S2F). Together, these data suggest that NRF1 promotes HCC cell growth by inducing G1-S cell cycle transition.

### NRF1 facilitates migration and invasion of HCC cells by regulating epithelial -mesenchymal transition

**Fig. 3:**
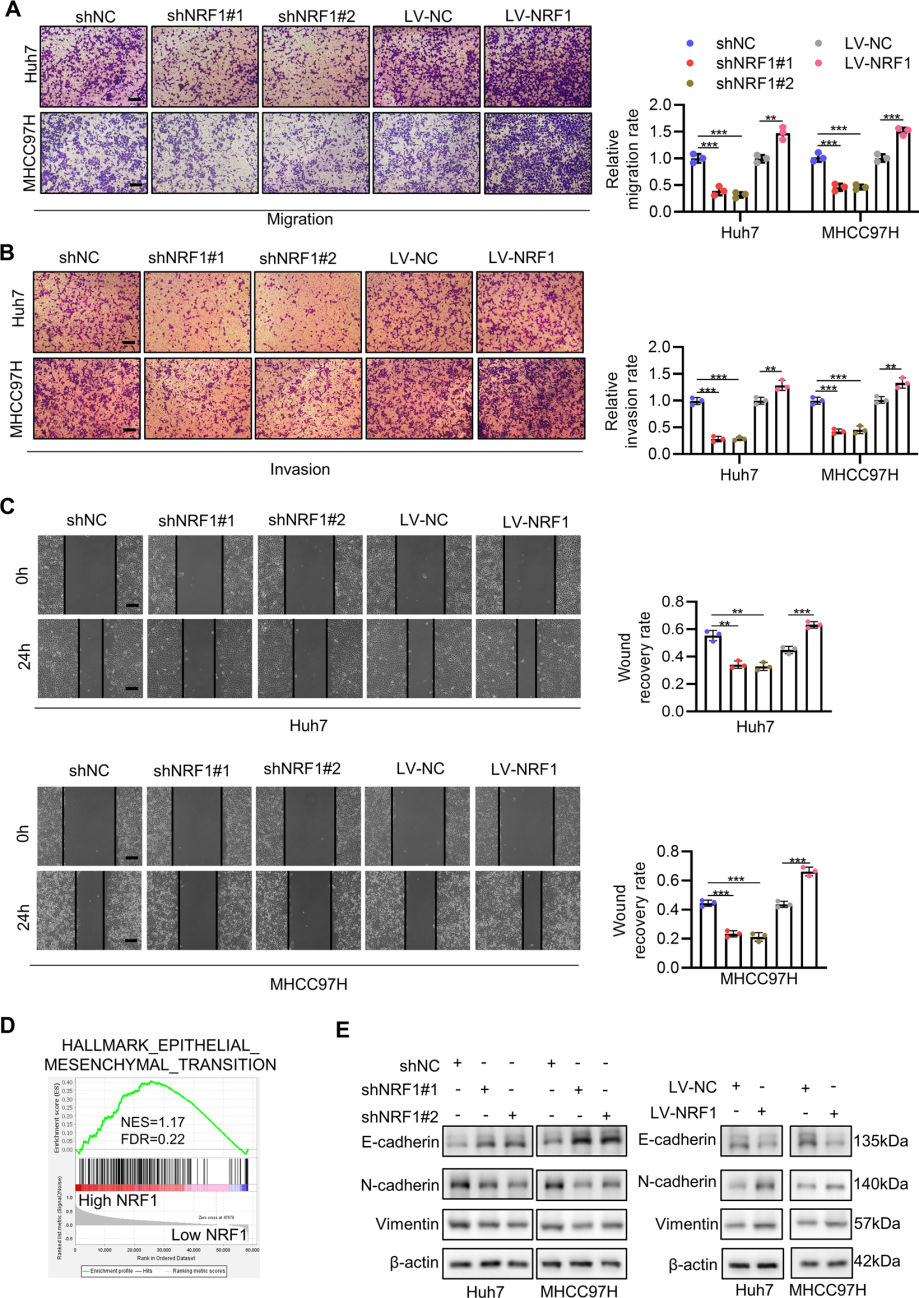
NRF1 facilitates migration and invasion of HCC cells by regulating epithelial-mesenchymal transition. **A**-**B** Huh7 and MHCC97 cell migration (**A**) and invasion (**B**) ability were analysed by transwell assay after NRF1 knockdown or overexpression. Scale bar: 100 μm. The bar graph showed the relative cell number. **C** Huh7 and MHCC97H cell migration ability was analysed by wound healing assay after NRF1 knockdown or overexpression. Scale bar: 100 μm. The bar graphs showed the quantitative wound healing ability. **D** GSEA using EMT gene set between high NRF1 and low NRF1 group in TCGA-LIHC dataset. **E** Western blot showed the EMT related protein levels in Huh7 and MHCC97H after NRF1 knockdown or overexpression. *p < 0.05, ** p  < 0.01, *** p  < 0.001

As metastasis is the main reason of HCC related deaths, we then studied the effects of NRF1 on the migration and invasion ability of HCC cells. Transwell migration assay revealed that knockdown of NRF1 strongly inhibited the migration of HCC cells, while overexpression of NRF1 showed the opposite effects (Fig. 3A). Transwell invasion assay exhibited similar results (Fig. 3B). In line with this, wound healing assay indicated that knockdown of NRF1 significantly inhibited the wound healing ability of Huh7 and MHCC97H cell while NRF1 overexpression facilitated the wound healing (Fig. 3C). Since epithelial-mesenchymal transition (EMT) is a critical process for the invasion and metastasis of tumor cells, we wondered whether NRF1 could affect EMT of HCC cells. Coincidently, by performing GSEA using TCGA-LIHC data, we found EMT-associated genes were significantly enriched in high NRF1 group (Fig. 3D). Thus, we utilized Western blot to detect the epithelial and mesenchymal markers. The result demonstrated that NRF1 knockdown decreased the protein level of the mesenchymal markers N-cadherin and Vimentin but increased that of the epithelial marker E-cadherin, while NRF1 overexpression exhibited the converse effect (Figs. 3E and S3A-S3C). Taken together, these results indicate that NRF1 facilitates migration and invasion of HCC cells by regulating epithelial -mesenchymal transition.

### LPCAT1 is a direct transcriptional target of NRF1

**Fig. 4:**
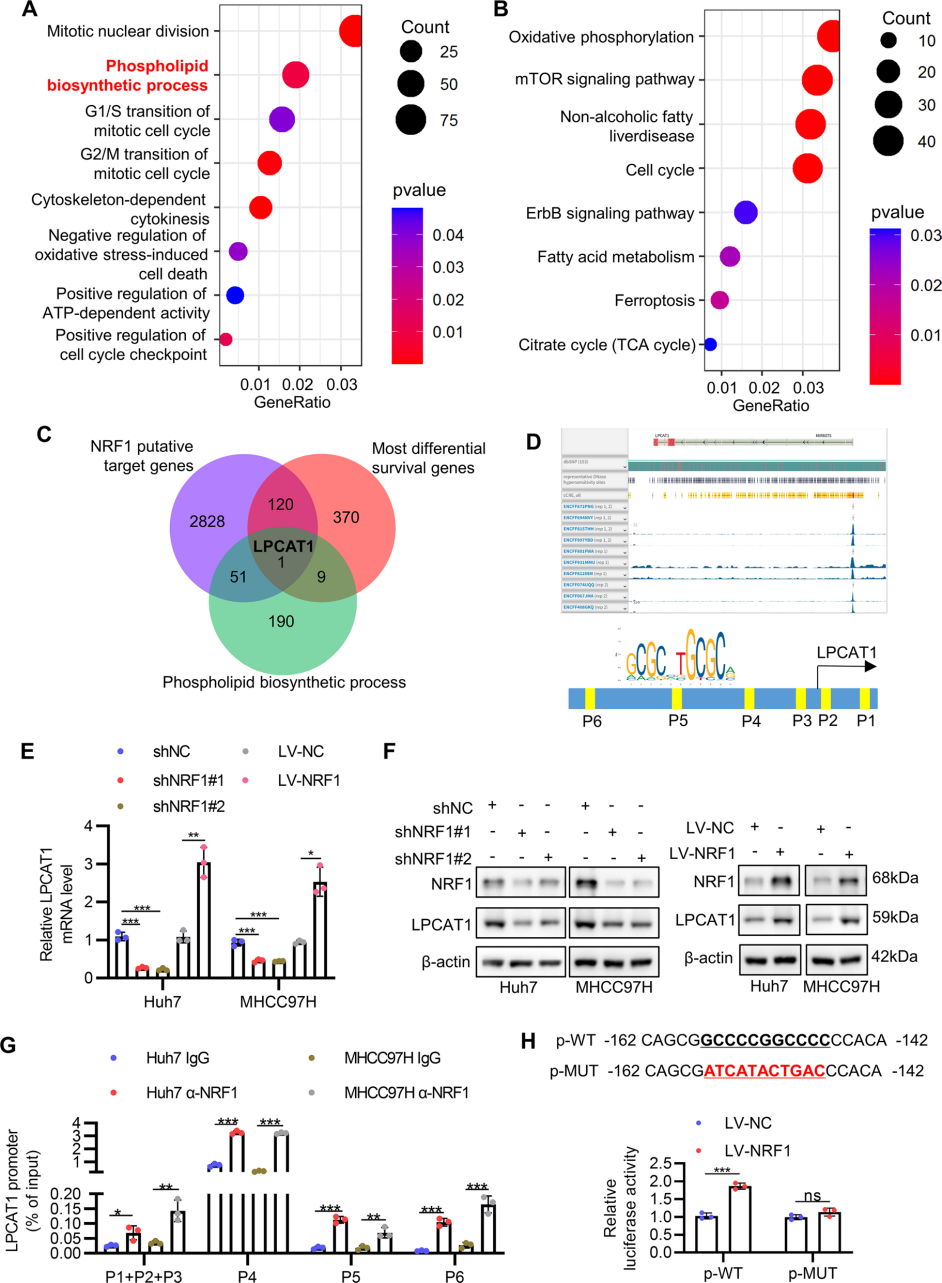
LPCAT1 is a direct transcriptional target of NRF1. **A**-**B** NRF1 function was inferred by GO (**A**) and KEGG (**B**) enrichment analysis with top 3000 putative NRF1 target genes. **C** Venn plot showed the intersection of the three gene sets (top 3000 NRF1 putative target genes, top 500 most differential survival genes in HCC and phospholipid biosynthetic process gene set). (**D**) Upper: screenshot from ENCODE website showing the NRF1 binding peak in ChIP-seq experiment near LPCAT1 transcription start site. Lower: the putative binding sites of NRF1 on LPCAT1 promoter predicted by JASPAR website was shown (P1:55 ~ 65;P2:9 ~ 19;P3:-16 ~ -6;P4:-157 ~ -147;P5:-324 ~ -314;P6:-695 ~ -685). **E**–**F** The mRNA and protein levels of LPCAT1 in Huh7 and MHCC97H after NRF1 knockdown or overexpression were determined with qRT-PCR (**E**) and Western blot (**F**), respectively. **G** ChIP-qPCR assay was performed to analyse the binding of NRF1 on LPCAT1 promoter. α-NRF1, anti-NRF1 antibody. **H** Dual luciferase reporter assay showed the relative LPCAT1 promoter luciferase activity in Huh7 cells after NRF1 overexpression. *p < 0.05, ** p  < 0.01, *** p  < 0.001

Since the above experiments showed NRF1 played an oncogenic role in HCC cells, we sought to explore the underlying mechanism. The expression of PGC1-α (a transcription coactivator indispensable in mitochondrial biogenesis) and mtDNA content both decreased in HCC compared with adjacent normal tissue,[17, 18] suggesting that NRF1 promotes HCC progression through mechanisms other than mitochondrial biogenesis. Therefore, we initialized our investigation by analysing its transcriptional targets. We performed GO and KEGG enrichment analysis focusing on the top 3000 putative NRF1 target genes downloaded from chip-atlas website to overview the NRF1 transcriptional function. As shown in Fig. 4A and B, cell cycle, oncogenic signaling and mitochondrial metabolism related genes were significantly enriched in NRF1 target genes, which was in accordance with previous studies.[3, 19] Surprisingly, we noticed ‘Phospholipid biosynthetic process’ was enriched in GO enrichment analysis (Fig. 4A). Considering that persistent lipid biosynthesis is prerequisite for tumor cell proliferation,[20] we took interest in this finding and conducted further investigation. In search of the most significant genes that mediate the oncogenic effect of NRF1, we used Venn analysis to find the intersection of the top 3000 putative NRF1 target genes (Additional file 12: Table S4), the top 500 most differential survival genes in HCC (Additional file 13: Table S5) and phospholipid biosynthetic process gene set (Additional file 14: Table S6) (Fig. 4C). Interestingly, there was only one gene in the intersection, which was LPCAT1, a phospholipid remodeling enzyme that was reported to promote HCC malignant transformation previously.[14, 16] Therefore, we focused on LPCAT1 in the following investigation. We found a sharp peak exactly at the LPCAT1 transcription start site (TSS) in NRF1 ChIP-seq data that was conducted in HepG2 cell line (Fig. 4D) on ENCODE website.[21] JASPAR website also predicted six NRF1 consensus sequence in the promoter region of LPCAT1 gene (Fig. 4D).[22] These results highly suggested that LPCAT1 was a direct target of NRF1. To validate this hypothesis, we detected the mRNA level of LPCAT1 by qRT-PCR after silencing or overexpressing NRF1. As expected, both in Huh7 and MHCC97H, NRF1 knockdown dramatically downregulated LPCAT1 mRNA level, while overexpression of NRF1 displayed the opposite results (Fig. 4E). Consistently, the result of protein level as revealed by Western blot was the same (Figs. 4F, S3D, S3E). We then conducted ChIP-qPCR assay to evaluate the binding of NRF1 to the promoter of LPCAT1. Due to the highly repetitive sequences near LPCAT1 TSS, we failed to design primers specifically expanding each of P1/P2/P3 sites, so we detected them as a whole. The binding of NRF1 to the six predicted NRF1 binding sites were all significantly higher than the IgG control, with P4 site showing the highest binding capacity (Fig. 4G). Furthermore, the luciferase activity that was driven by wild-type LPCAT1 promoter was substantially enhanced by NRF1 overexpression in Huh7 (Fig. 4H) and was tremendously attenuated by NRF1 knockdown in MHCC97H (Figure S3F). As P4 site showed the highest binding capacity for NRF1, we mutated this site in luciferase reporter plasmid, which nearly completely abolished the regulation of NRF1 on LPCAT1 promoter activity (Figs. 4H and S3F). Taken together, these results demonstrate that LPCAT1 is a direct transcriptional target of NRF1.

### LPCAT1 and NRF1 constitute a positive feedback loop through activating the ERK1/2-CREB pathway

**Fig. 5:**
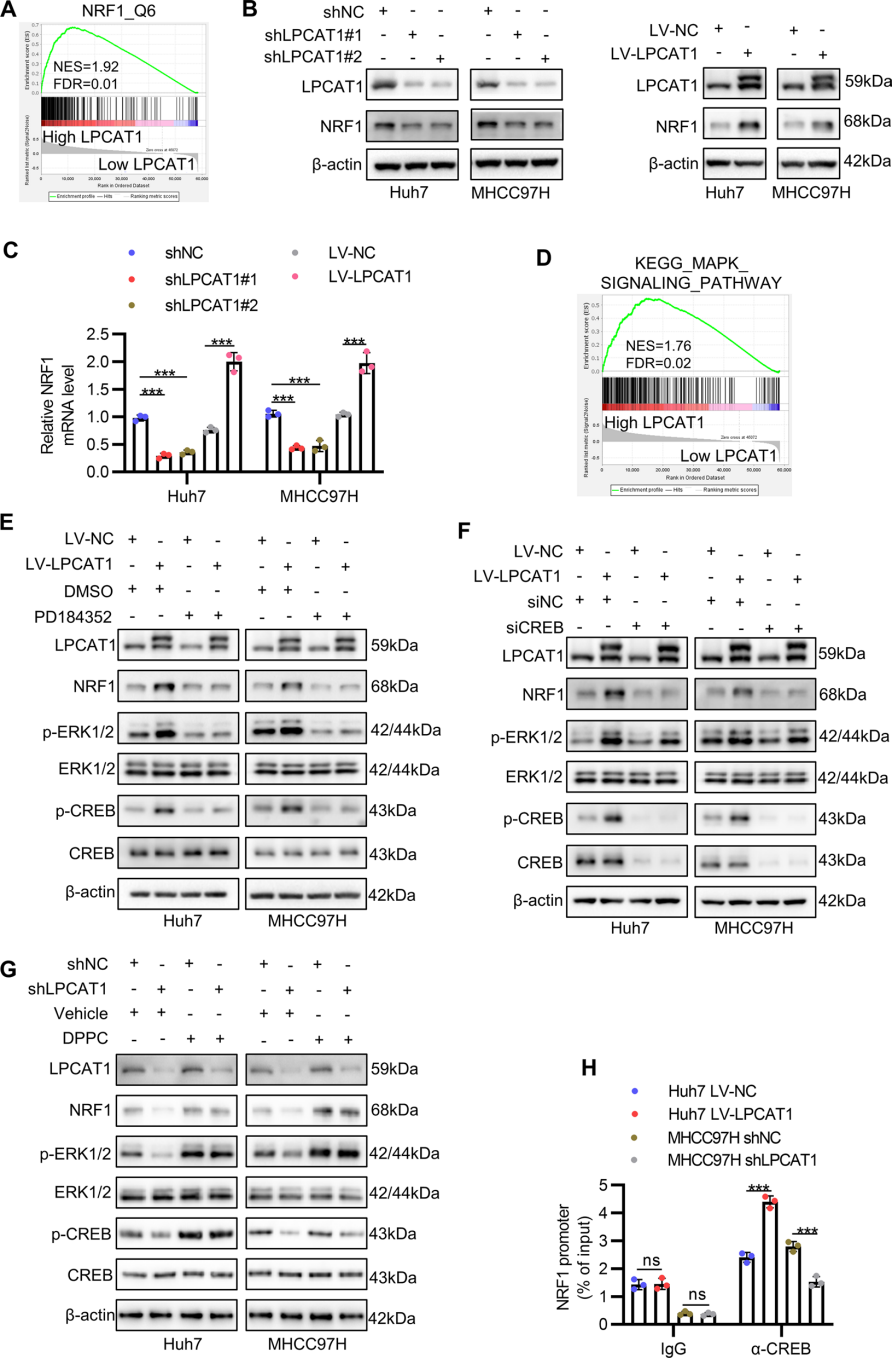
LPCAT1 and NRF1 constitute a positive feedback loop through activating the ERK1/2-CREB pathway. **A** NRF1 activity difference was analysed between high LPCAT1 and low LPCAT1 group with GSEA in TCGA-LIHC dataset. **B**-**C** NRF1 protein and mRNA levels in Huh7 and MHCC97H after LPCAT1 overexpression or knockdown were determined by Western blot (**B**) and qRT-PCR (**C**), respectively. **D** GSEA was performed to analyse MAPK activation in high and low LPCAT1 expression group. **E**–**F** The activation of ERK1/2-CREB pathway and NRF1 protein levels after LPCAT1 overexpression and PD184352 treatment (**E**) or CREB knockdown (**F**) were analysed by Western blot. **G** The activation of ERK1/2-CREB pathway and NRF1 protein levels after LPCAT1 knockdown and DPPC supplementation were analysed by Western blot. **H** ChIP-qPCR was applied to analyse the binding of CREB on NRF1 promoter after LPCAT1 knockdown or overexpression. α-CREB, anti-CREB antibody. *p < 0.05, ** p  < 0.01, *** p  < 0.001

To explore how LPCAT1 functioned in HCC, we conducted GSEA once more utilizing TCGA-LIHC transcriptome profile. Unexpectedly, in comparing the gene expression difference between high LPCAT1 and low LPCAT1 group, we found that gene set NRF1_Q6 was significantly enriched in high LPCAT1 group (Fig. 5A), which suggested that LPCAT1 enhanced NRF1 transcriptional activity. To determine whether it was because LPCAT1 increased the expression level of NRF1, we performed Western blot and the result indicated that LPCAT1 knockdown decreased NRF1 protein level in both Huh7 and MHCC97H and vice versa (Figs. 5B, S3G, S3H). Next, the qRT-PCR result showed that NRF1 mRNA level displayed the same trend as protein level, which further demonstrated that LPCAT1 regulated NRF1 expression on transcriptional level (Fig. 5C). GSEA using KEGG gene sets also indicated that MAPK signaling pathway was significantly enriched in high LPCAT1 group (Fig. 5D). Meanwhile, previous study reported that ERK1/2 positively regulates CREB phosphorylation on Ser133,[23] and that CREB directly transactivates NRF1.[5] Based on these findings, we hypothesized that LPCAT1 might regulate NRF1 expression through ERK1/2-CREB signaling. Indeed, LPCAT1 overexpression increased the protein level of NRF1 and the phosphorylation of ERK1/2 and CREB in Huh7 and MHCC97H, which were abrogated by administration of PD184352, a selective MEK1/2 inhibitor which impeded the phosphorylation of ERK1/2 by MEK1/2 (Fig. 5E and S4A-S4D). Similarly, a siRNA specifically targeting CREB decreased the total CREB and p-CREB, as well as NRF1 protein levels after LPCAT1 overexpression (Fig. 5F and S4E-S4H). Consistently, PD184352 and CREB knockdown both significantly reversed the elevation of NRF1 mRNA induced by LPCAT1 overexpression (Figures S5A and S5B). We then sought to understand the mechanism by which LPCAT1 activated ERK1/2-CREB signaling pathway and NRF1 transcription. Because the main metabolite of LPCAT1 is DPPC, which is a kind of saturated PC that can facilitate oncogenic signaling transduction from cell membrane to cytosol by forming lipid rafts on the cell membrane,[15] we speculated that DPPC mediated the activation of ERK1/2-CREB signaling induced by LPCAT1. Therefore, we conducted rescue experiments with concomitant LPCAT1 knockdown and exogenous DPPC supplementation. As expected, knockdown of LPCAT1 decreased activation level of ERK1/2-CREB pathway and expression level of NRF1, which was reversed by DPPC administration (Fig. 5G and S5C-S5G). In addition, ChIP-qPCR using anti-CREB antibody revealed that overexpression of LPCAT1 enhanced the binding of CREB to NRF1 promoter in Huh7 and knockdown of LPCAT1 inhibited that in MHCC97H, while ChIP-qPCR using IgG control had no such change (Fig. 5H). Together, these results indicate that LPCAT1 and NRF1 constitute a positive feedback loop through activating the ERK1/2-CREB pathway.

### LPCAT1 mediates the oncogenic potential of NRF1 by modulating the ERK1/2-CREB signaling

**Fig. 6:**
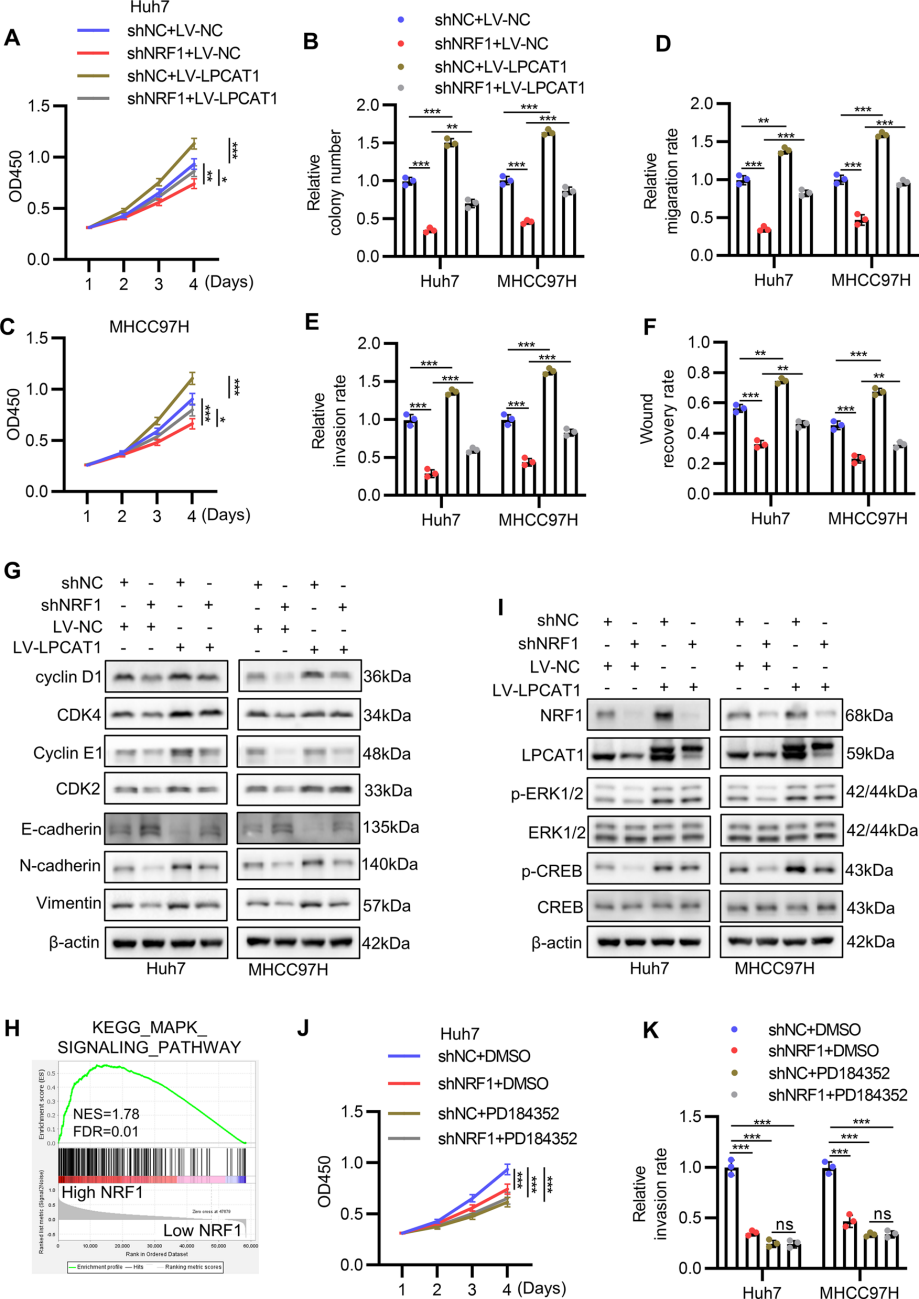
LPCAT1 mediates the oncogenic potential of NRF1 by modulating the ERK1/2-CREB signaling. **A**-**C** The proliferation ability of Huh7 and MHCC97H cells after NRF1 knockdown and LPCAT1 overexpression were analysed by CCK8 (**A**-**B**) and colony formation assay (**C**). (**D**-**F**) The migration and invasion ability of Huh7 and MHCC97H cells after NRF1 knockdown and LPCAT1 overexpression were analysed by transwell (**D**-**E**) and wound healing assay (**F**). (**G**) The cell cycle and EMT process related genes expression were determined by Western blot in Huh7 and MHCC97H after NRF1 knockdown and LPCAT1 overexpression. **H** GSEA using KEGG MAPK pathway gene set between high NRF1 group and low NRF1 group in TCGA-LIHC dataset. **I** The activation of ERK1/2-CREB pathway and NRF1, LPCAT1 protein levels were analysed by Western blot after NRF1 knockdown and LPCAT1 overexpression. **J** The proliferation ability of Huh7 cells after NRF1 knockdown and PD184352 treatment was determined by CCK8 assay. **K** The invasion ability of Huh7 and MHCC97H cells after NRF1 knockdown and PD184352 treatment was determined by transwell assay. *p < 0.05, **p < 0.01, ***p < 0.001, ns, not significant

LPCAT1 was reported to promote HCC proliferation and invasion.[16] Thus, we wondered whether NRF1 promotes proliferation and invasion through LPCAT1 induction. Therefore, we silenced NRF1 and overexpressed LPCAT1 in Huh7 and MHCC97H and performed functional assay. As expected, results of CCK8 assay and colony formation assay indicated that LPCAT1 overexpression reversed the inhibition of NRF1 knockdown on HCC cell proliferation (Fig. 6A-C). Transwell and wound healing assay also demonstrated that LPCAT1 overexpression rescued the inhibition of shNRF1 on HCC cell migration and invasion (Fig. 6D-F). Consistently, Western blot revealed that LPCAT1 overexpression abrogated the effect of NRF1 knockdown on cell cycle and EMT related protein expression (Figs. 6G, S6A-S6G). We also found that GSEA using KEGG gene sets suggested that MAPK signaling pathway was enriched in high NRF1 group compared with low NRF1 group (Fig. 6H), implying that NRF1 might also influence ERK1/2 activation. Based on the aforementioned findings that NRF1 induces LPCAT1 transcription and LPCAT1 activates ERK1/2-CREB signaling pathway, we wondered if NRF1 activates ERK1/2-CREB signaling pathway through LPCAT1. As shown in Fig. 6I and S7A-S7D, knockdown of NRF1 downregulated p-ERK1/2 and p-CREB levels which was rescued by concomitant LPCAT1 overexpression. Since ERK1/2 and CREB promoted HCC tumorigenesis,[24–26] we conducted rescue experiments. Predictably, CCK8 and transwell invasion assay indicated that both NRF1 knockdown and PD184352 significantly inhibited proliferation and invasion capability of HCC cell but NRF1 knockdown and PD184352 had no additive effects (Fig. 6J and K), suggesting ERK1/2 signaling pathway was a downstream effector of NRF1. Rescue experiments using CREB siRNA showed similar results (Figures S7E and S7F). Altogether, these results indicate that LPCAT1 mediates the oncogenic potential of NRF1 by modulating the ERK1/2-CREB signaling.

### NRF1 promotes HCC progression in vivo

**Fig. 7:**
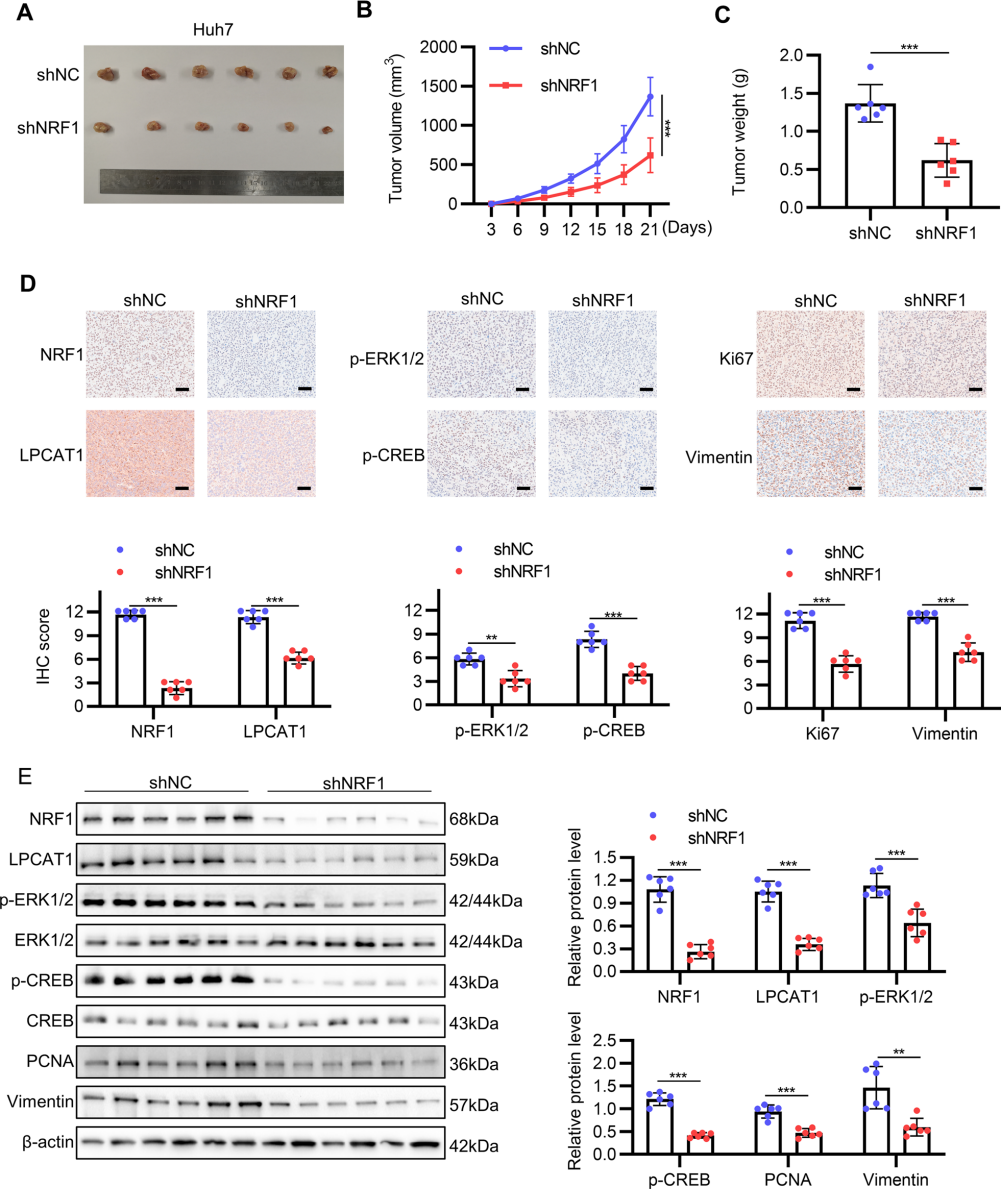
NRF1 promotes HCC growth in vivo. Lentivirus was used to establish stable NRF1 knockdown Huh7 cell lines and tumor growth ability was assessed with subcutaneous xenograft model. **A** The image of subcutaneous xenograft tumors were shown. **B** The tumor volume was measured every three days. **C** The mass of the subcutaneous xenograft tumors was weighed after excision. **D** Representative images of IHC staining and the IHC scores of NRF1, LPCAT1, p-ERK1/2, p-CREB, Ki67 and Vimentin of the tumors. Scale bar: 50 μm. **E** NRF1, LPCAT1, p-ERK1/2, ERK1/2, p-CREB, CREB, PCNA and Vimentin were analyzed with Western blot. Bar graphs showed the quantitative results. *p < 0.05, **p < 0.01, ***p < 0.001

Finally, we examined the effect of silencing NRF1 on tumor progression in vivo. To evaluate the effect of NRF1 on tumor growth in vivo, Huh7 cells that were infected with NRF1 knockdown lentivirus and control lentivirus were utilized to construct subcutaneous xenograft tumor model. Similar to the in vitro results, we found that the tumor volumes in shNRF1 group were significantly smaller than shNC group (Fig. 7A and B), and tumor weights were also lower upon NRF1 knockdown (Fig. 7C). Consistently, IHC results showed that there was less NRF1, LPCAT1, p-ERK1/2, p-CREB, Ki67 and Vimentin staining in NRF1 knockdown group (Fig. 7D), which was further validated by Western blot results (Fig. 7E).

**Fig. 8:**
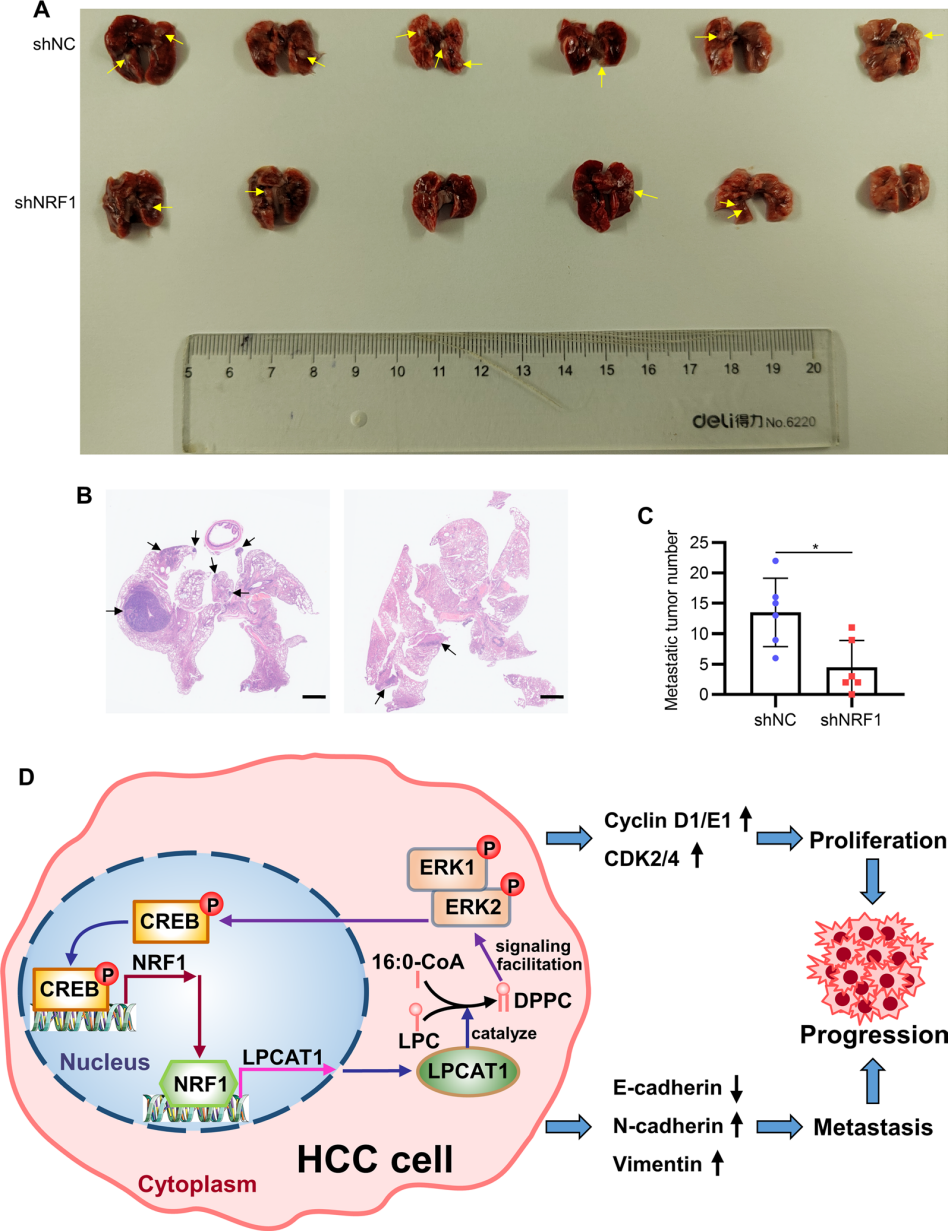
NRF1 promotes HCC metastasis in vivo. Lentivirus was used to establish stable NRF1 knockdown MHCC97H cell lines and tumor metastasis ability was assessed with tail vein injection model. **A** Lungs of the nude mice were excised and photographed. Arrows indicate metastasis loci. **B** Representative images of HE staining of the lungs to evaluate metastasis nodes. Arrows indicate metastasis loci. Scale bars: 1 mm. **C** The number of quantitative results of lung metastasis tumor. **D** The schematic of the present study. *p < 0.05, **p < 0.01, ***p < 0.001

We then assessed whether NRF1 could drive tumor metastasis in vivo. Stable NRF1 knockdown MHCC97H cells were injected through tail vein and six weeks later the lung metastasis nodules were examined. As shown in Fig. 8A-C, photographs and HE staining both revealed that silencing NRF1 impeded tumor cell metastasis to the lung. In summary, these results suggest that NRF1 promotes HCC progression in vivo. Based on these findings, we detected LPCAT1 protein expression level in our HCC tissue microarray and found that LPCAT1 protein level showed significant positive correlation with NRF1 protein level (Figure S7G). The schematic diagram of the present study is shown in Fig. 8D.

## Discussion

In the present study, we found that NRF1 promotes the proliferation and migration of HCC cells. NRF1 is overexpressed in HCC and its target genes are also upregulated in HCC, which suggest that NRF1 function is overactive in HCC tumorigenesis. Besides, NRF1 expression increases with the TNM stage and pathological grade, and high NRF1 indicates poor overall survival and disease free survival. In vitro and in vivo experiments indicated silencing NRF1 inhibits proliferation and invasion of HCC, which further consolidated its oncogenic role.

NRF1 is well-known for its function of regulating mitochondria biogenesis by controlling the transcription of TFAM, cytochrome c and other proteins necessary for mitochondria.[3] Accumulating evidence supports that NRF1 participates in the progression of several types of cancer. NRF1 regulates CXCR4 expression and modulates redox status which contribute to the pathogenesis of breast cancer.[9, 27] Besides acting as an oncogene in breast cancer,[6, 7] NRF1 also facilitates tumor progression in several other cancers. NRF1 is associated with severity of astrocytoma and indicates poor prognosis of glioblastoma [11]. Also, it promotes proliferation of renal cancer cells by regulating TFE3.[10] However, studies also reported that NRF1 might inhibit tumor progression in some specific cancer types. NRF1 suppresses TGF-β signaling and inhibits the proliferation and migration of PC3 prostate cancer cells.[12] Besides, NRF1 regulates the expression of autophagy related 5 (ATG5) and autophagy related 7 (ATG7) and inhibits the migration of melanoma cells.[13] Therefore, the function of NRF1 is complex and may play different roles in tumor progression depending on specific tumor types. Our present work highlighted the oncogenic role of NRF1 in HCC with evidence from both bioinformatic analysis and in vitro or in vivo experiments.

Mechanistically, we discovered NRF1 directly activates the transcription of LPCAT1, an enzyme remodeling the side chain of phospholipid. After PC are de novo synthesized, their fatty acyl chains at the sn-2 site are hydrolyzed to produce lysophosphatidylcholine (LPC) which are then reacylated by LPCAT to incorporate another fatty acid to the sn-2 position and form a new PC species, which is called Land’s cycle.[14] Since PC constitute the majority of plasma membrane and other biological membrane, cell membranes undergo dynamic change to adapt to environmental change. LPCAT1 prefers 16:0-CoA (also known as palmitoyl-Co-A) as its substrate and its main catalytic product is DPPC. Overexpression of LPCAT1 increase DPPC and thus upregulate the plasma membrane PC saturation which facilitate signal transduction from membrane to cytosol.[15] As continuous signaling transduction like PI3K/Akt pathway, NF-κB pathway, and MAPK pathway are fundamental drivers of HCC tumorigenesis,[15] it’s well-reasoned that LPCAT1 promotes the malignant transformation of HCC. Despite its indubitable oncogenic role in various tumors, little is known about the mechanisms for its upregulation, except for several reports that specific microRNAs target LPCAT1.[28, 29] We find LPCAT1 is under direct control of NRF1, thus provide the evidence of transcriptional regulation of LPCAT1 for the first time. We did not analyze lipidomic change by mass spectrometry upon silencing NRF1 in our investigation, but considering the unequivocal regulation of NRF1 on LPCAT1 transcription and the explicit effect of LPCAT1 on PC metabolism,[16, 30, 31] we believe NRF1 could alter phospholipid metabolism in HCC.

Hyperactive ERK1/2 signaling pathway is a fundamental driving factor of HCC. Interestingly, activating mutations in RAS and RAF genes, which are common genetic events that activate ERK1/2 pathway in other cancers, are rarely found in HCC.[32] Therefore, the continuous activation of ERK1/2 may rely more on external regulatory factors than internal genetic alterations. ERK1/2 is known to enhance proliferation of cancer and represents a promising target in cancer treatment.[33] It may also facilitate EMT of HCC according to the latest reports.[25, 34] In the present study, we found NRF1-LPCAT1 axis could positively regulate ERK1/2 activation and thus promote proliferation and invasion of HCC, which further deepened our understanding of upstream factors regulating ERK1/2 in HCC.

CREB is a transcription factor that accelerates HCC progression.[24, 26] CREB could be activated by multiple kinases such as MAPK, Akt, PKA, PKC.[35] In our work, we found CREB phosphorylation on Ser133 is dramatically decreased by MEK1/2 inhibitor PD184352, which suggested that ERK1/2 controls CREB activation to a large extent in HCC, the same as a previous article mentioned.[23] Moreover, NRF1 promotes progression of HCC by activating CREB phosphorylation.

We found CREB directly regulates NRF1 transcription in HCC. This is in accordance with previous study.[5] which showed that inflammation induces NRF1 expression in liver cells through transcription factor NF-κB and CREB. Interestingly, we found besides PD184352, administration of PI3K inhibitor LY294002 or NF-κB inhibitor BAY 11–7082 both dramatically decrease NRF1 protein level in Huh7 and MHCC97H (Figure S8A). Therefore, NRF1 is under control of PI3K/Akt pathway, NF-κB pathway, and ERK1/2 pathway in HCC. As all of the three signaling pathway tend to be highly active in HCC, it’s well-reasoned that NRF1 is aberrantly overexpressed. NRF1 is not only activated by CREB but also activates multiple significant pathways in cancer (Figures S8A and S8B), which suggests that NRF1 plays a central role in HCC progression.

There are several limitations regarding our work. First, our results are seemingly contradictory with a previous report,[36] which implied that NRF1 suppresses HCC. In Wang CQ et al.’s study, the suppressive role of PGC1α and MPC1 on HCC were exhibited, while NRF1 was only emphasized as the cooperating transcription factor of PGC1α in regulating MPC1 transcription and its effect on HCC were not mentioned. Although we provide evidence that NRF1 promotes HCC progression through LPCAT1-ERK1/2-CREB axis, we could not rule out the possibility that NRF1 may inhibit HCC progression through some specific mechanisms under some specific conditions and it’s our opinion that the overall effect of NRF1 on HCC is promotive. While NRF1 is known to collaborate with transcription coactivator PGC1α to regulate target gene transcription in mitochondrial biogenesis, it’s interesting that PGC1α plays a suppressive role in HCC but NRF1 plays a promotive role.[37, 38] Considering that NRF1 can also act as a transcription cofactor of SMAD4 and that PGC1α also functions as transcription coactivator of ERRα, PPARγ etc.,[39] we hypothesize that in HCC the specific transcription pattern makes NRF1 and PGC1α not functionally integrated, which leads to the final difference in their function. Second, NRF1 induces mitochondrial biogenesis and mitochondria could affect tumorigenesis in a complex and profound manner as critical cellular processes are related to mitochondria, such as oxidative phosphorylation, ATP production, ROS generation and apoptosis.[40] Our present study highlights NRF1 facilitates HCC progression by inducing LPCAT1 transcription, but whether NRF1-mediated mitochondrial biogenesis plays a role in HCC deserves further exploration. Third, NRF1 could be phosphorylated and the phosphorylation promotes its nuclear translocation and activity of transactivation.[41, 42] Whether NRF1 is highly phosphorylated and to what extent its phosphorylation accounts for HCC progression still need to be investigated.

## Conclusion

In summary, we show NRF1 is overexpressed and overactive in HCC. It activates ERK1/2-CREB signaling pathway by inducing LPCAT1 transcription and thus promotes cell cycle progression and EMT of HCC cells. LPCAT1 induces NRF1 transcription reciprocally by activating ERK1/2-CREB signaling pathway, forming a regulation loop that continuously stimulates HCC progression. NRF1 is a promising therapeutic target of HCC.

### Supplementary Information

**Additional file 1** **Figure S1**. (A) Quantitative results of the protein levels after NRF1 expression manipulation. Related to Figure 2B. (B-E) The effect of NRF1 on mitochondrial function was analyzed through ATP level, glucose uptake, lactate secretion and NAD+/NADH ratio. *p<0.05, **p<0.01, ***p<0.001.

**Additional file 2** **Figure S2.** (A-D) Quantitative results of the protein levels after NRF1 expression manipulation. Related to Figure 2F. (E-F) The effect of NRF1 on cell apoptosis was analysed by flow cytometry. *p<0.05, **p<0.01, ***p<0.001.

**Additional file 3** **Figure S3**. (A-E) Quantitative results of the protein levels after NRF1 expression manipulation. Related to Figure 3E and Figure 4F. (F) Dual luciferase reporter assay showed the relative LPCAT1 promoter luciferase activity in MHCC97H cells after NRF1 knockdown. (G-H) Quantitative results of the protein levels after LPCAT1 expression manipulation. Related to Figure 5B. *p<0.05, **p<0.01, ***p<0.001.

**Additional file 4** **Figure S4**. (A-D) Quantitative results of the protein levels after LPCAT1 overexpression and PD184352 administration. Related to Figure 5E. (E-H) Quantitative results of the protein levels after LPCAT1 overexpression and CREB knockdown. Related to Figure 5F. *p<0.05, **p<0.01, ***p<0.001.

**Additional file 5** **Figure S5**. (A-B) NRF1 mRNA level was analyzed by qRT-PCR after LPCAT1 overexpression and PD184352 treatment (A) or CREB knockdown (B). (C-F) Quantitative results of the protein levels after LPCAT1 knockdown and DPPC supplementation. Related to Figure 5G. *p<0.05, **p<0.01, ***p<0.001.

**Additional file 6** **Figure S6**. (A-G) Quantitative results of the protein levels after NRF1 knockdown and LPCAT1 overexpression. Related to Figure 6G. *p<0.05, **p<0.01, ***p<0.001.

**Additional file 7 Figure S7**. (A-D) Quantitative results of the protein levels after NRF1 knockdown and LPCAT1 overexpression. Related to Figure 6I. (E-F) The proliferation and invasion ability after NRF1 and CREB knockdown were analyzed by CCK8 (E) and transwell assay (F). (G) Representative IHC results of NRF1 and LPCAT1 and their expression correlation in our HCC tissue microarray. Scale bar: 50 μm.

**Additional file 8 Figure S8**. (A) Western blot result of NRF1 protein level after LY294002 or BAY 11-7082 administration. (B) GSEA with KEGG pathways in cancer gene set between high NRF1 and low NRF1 group in TCGA-LIHC dataset. *p<0.05, **p<0.01, ***p<0.001

**Additional file 9 Table S1. **shRNA and siRNA sequences.

**Additional file 10 Table S2. **qRT-PCR primer sequences.

**Additional file 11 Table S3. **ChIP-qPCR primer sequences.

**Additional file 12 Table S4. **Top 3000 putative NRF1 target genes.

**Additional file 13 Table S5. **Top 500 most differential survival genes in HCC.

**Additional file 14 Table S6. **Phospholipid biosynthetic process gene set.

## Data Availability

All data used in this study is from open access websites described in materials and methods.

## Abbreviations

NRF1: Nuclear respiratory factor 1
HCC: Hepatocellular carcinoma
ChIP: Chromatin immunoprecipitation
LPCAT1: Lysophosphatidylcholine acyltransferase 1
ERK: Extracellular regulated protein kinases
CREB: CAMP-response element binding protein
EMT: Epithelial mesenchymal transition
TFAM: Mitochondrial transcription factor A
PC: Phosphatidylcholine
LPC: Lysophosphatidylcholine
DPPC: Dipalmitoyl phosphatidylcholine
GSEA: Gene set enrichment analysis
