# Supplementary figures and images for: Nuclear respiratory factor 1 drives hepatocellular carcinoma progression by activating LPCAT1-ERK1/2-CREB axis
Source: Biol Direct. 2023 Oct 24;18:67. doi: 10.1186/s13062-023-00428-z (PMC10594727; doi:10.1186/s13062-023-00428-z)

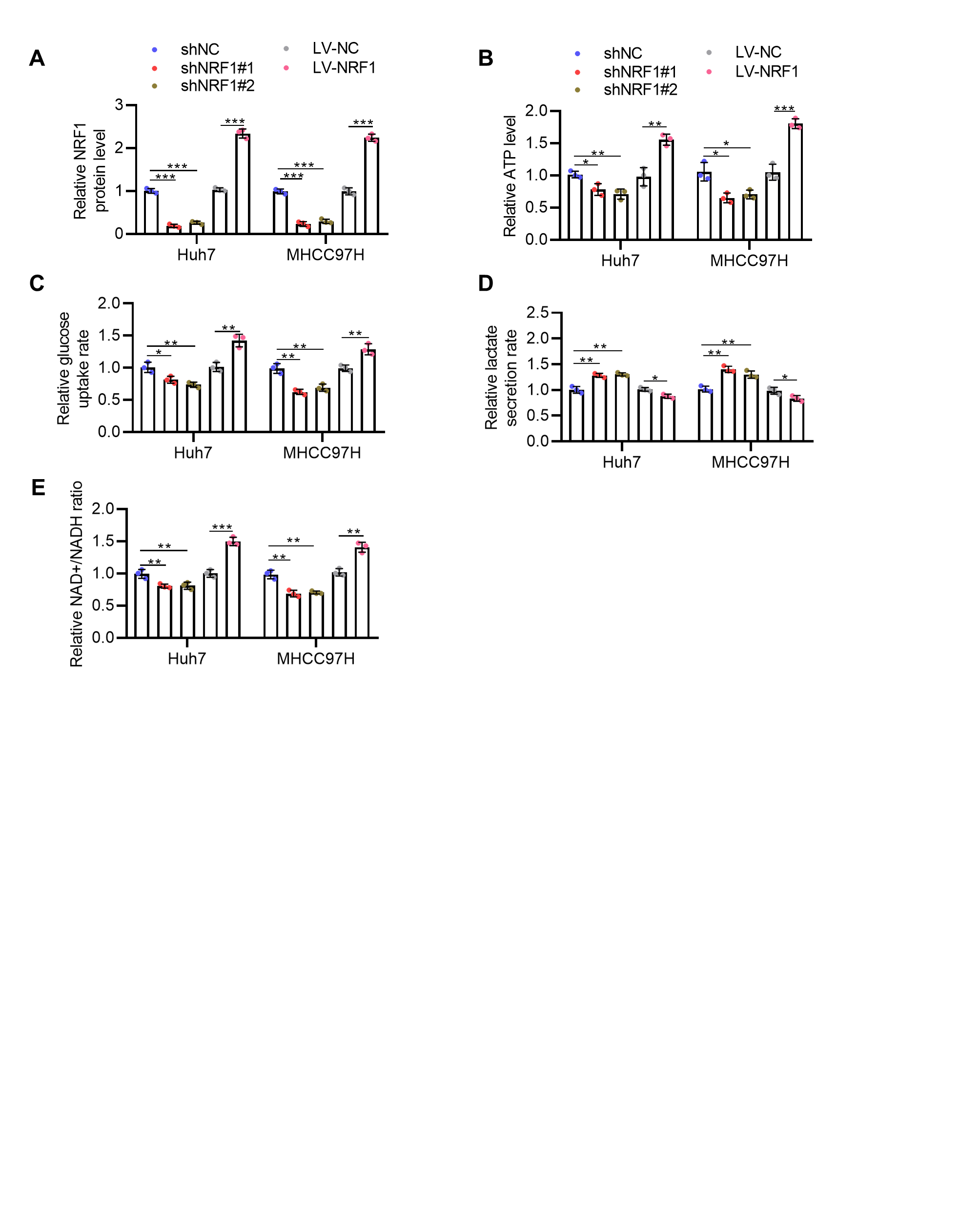

Supplement: Supplementary file 1 — Additional file 1 Figure S1. (A) Quantitative results of the protein levels after NRF1 expression manipulation. Related to Figure 2B. (B-E) The effect of NRF1 on mitochondrial function was analyzed through ATP level, glucose uptake, lactate secretion and NAD+/NADH ratio. *p<0.05, **p<0.01, ***p<0.001. [file 13062_2023_428_MOESM1_ESM.tif]

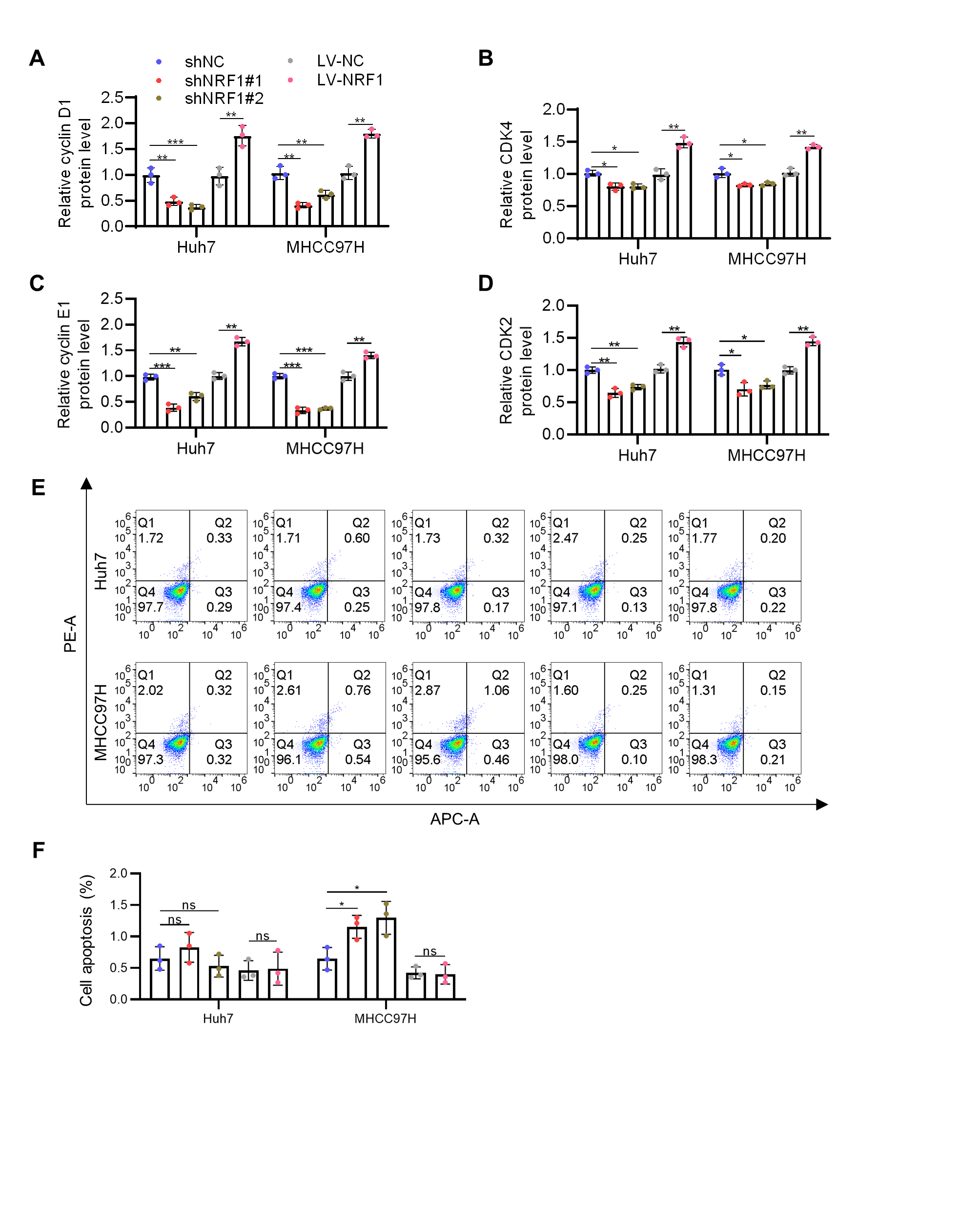

Supplement: Supplementary file 2 — Additional file 2 Figure S2. (A-D) Quantitative results of the protein levels after NRF1 expression manipulation. Related to Figure 2F. (E-F) The effect of NRF1 on cell apoptosis was analysed by flow cytometry. *p<0.05, **p<0.01, ***p<0.001. [file 13062_2023_428_MOESM2_ESM.tif]

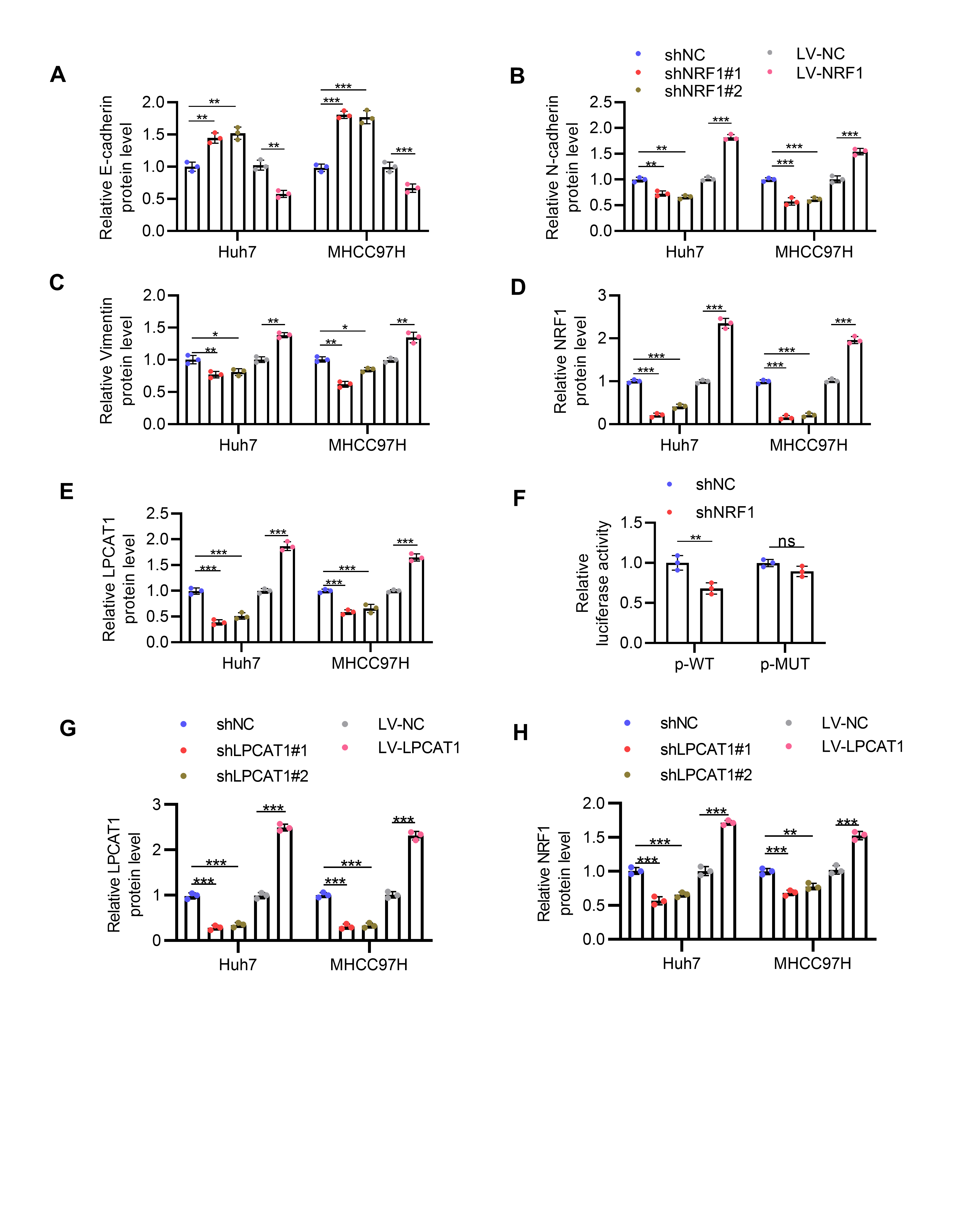

Supplement: Supplementary file 3 — Additional file 3 Figure S3. (A-E) Quantitative results of the protein levels after NRF1 expression manipulation. Related to Figure 3E and Figure 4F. (F) Dual luciferase reporter assay showed the relative LPCAT1 promoter luciferase activity in MHCC97H cells after NRF1 knockdown. (G-H) Quantitative results of the protein levels after LPCAT1 expression manipulation. Related to Figure 5B. *p<0.05, **p<0.01, ***p<0.001. [file 13062_2023_428_MOESM3_ESM.tif]

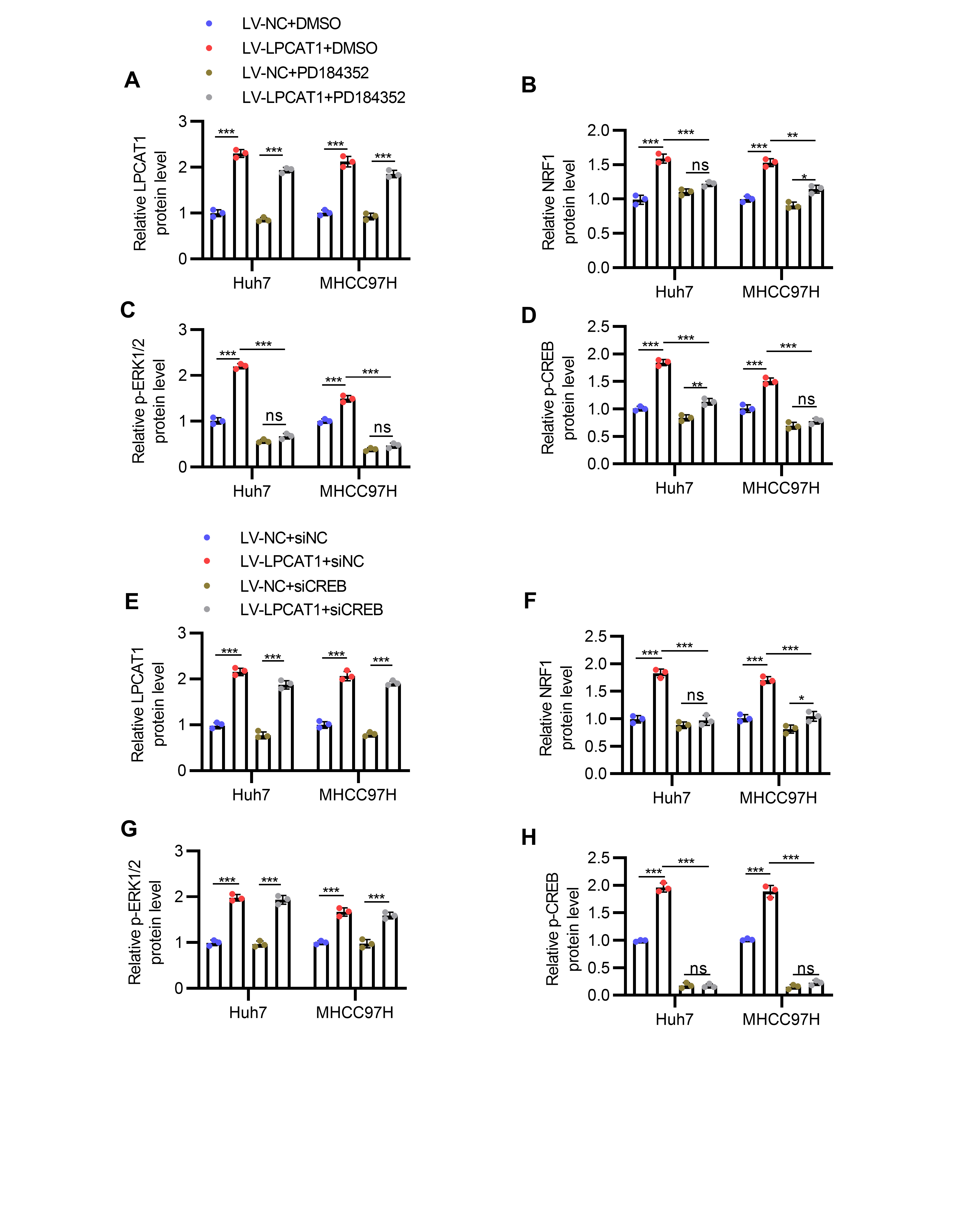

Supplement: Supplementary file 4 — Additional file 4 Figure S4. (A-D) Quantitative results of the protein levels after LPCAT1 overexpression and PD184352 administration. Related to Figure 5E. (E-H) Quantitative results of the protein levels after LPCAT1 overexpression and CREB knockdown. Related to Figure 5F. *p<0.05, **p<0.01, ***p<0.001. [file 13062_2023_428_MOESM4_ESM.tif]

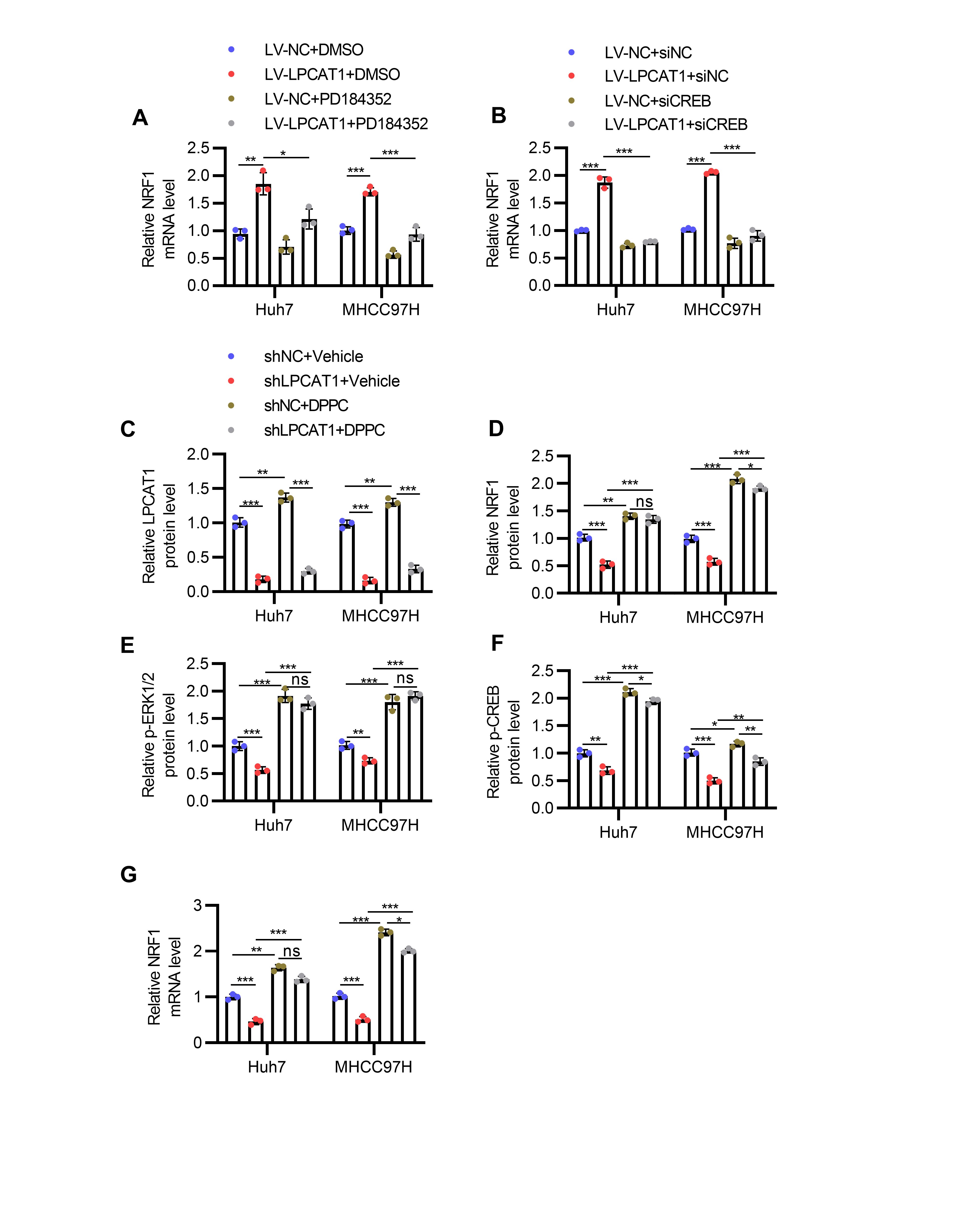

Supplement: Supplementary file 5 — Additional file 5 Figure S5. (A-B) NRF1 mRNA level was analyzed by qRT-PCR after LPCAT1 overexpression and PD184352 treatment (A) or CREB knockdown (B). (C-F) Quantitative results of the protein levels after LPCAT1 knockdown and DPPC supplementation. Related to Figure 5G. *p<0.05, **p<0.01, ***p<0.001. [file 13062_2023_428_MOESM5_ESM.tif]

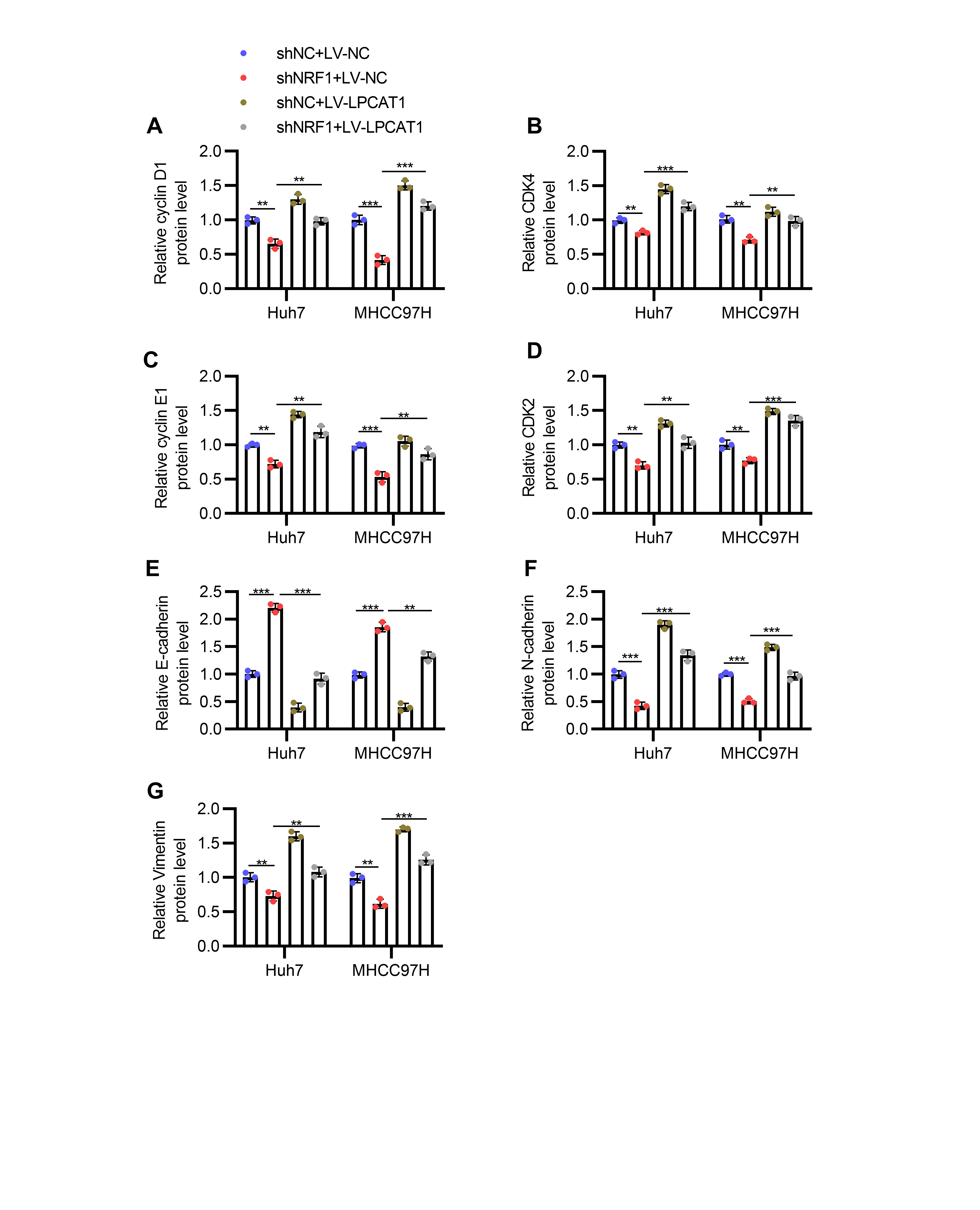

Supplement: Supplementary file 6 — Additional file 6 Figure S6. (A-G) Quantitative results of the protein levels after NRF1 knockdown and LPCAT1 overexpression. Related to Figure 6G. *p<0.05, **p<0.01, ***p<0.001. [file 13062_2023_428_MOESM6_ESM.tif]

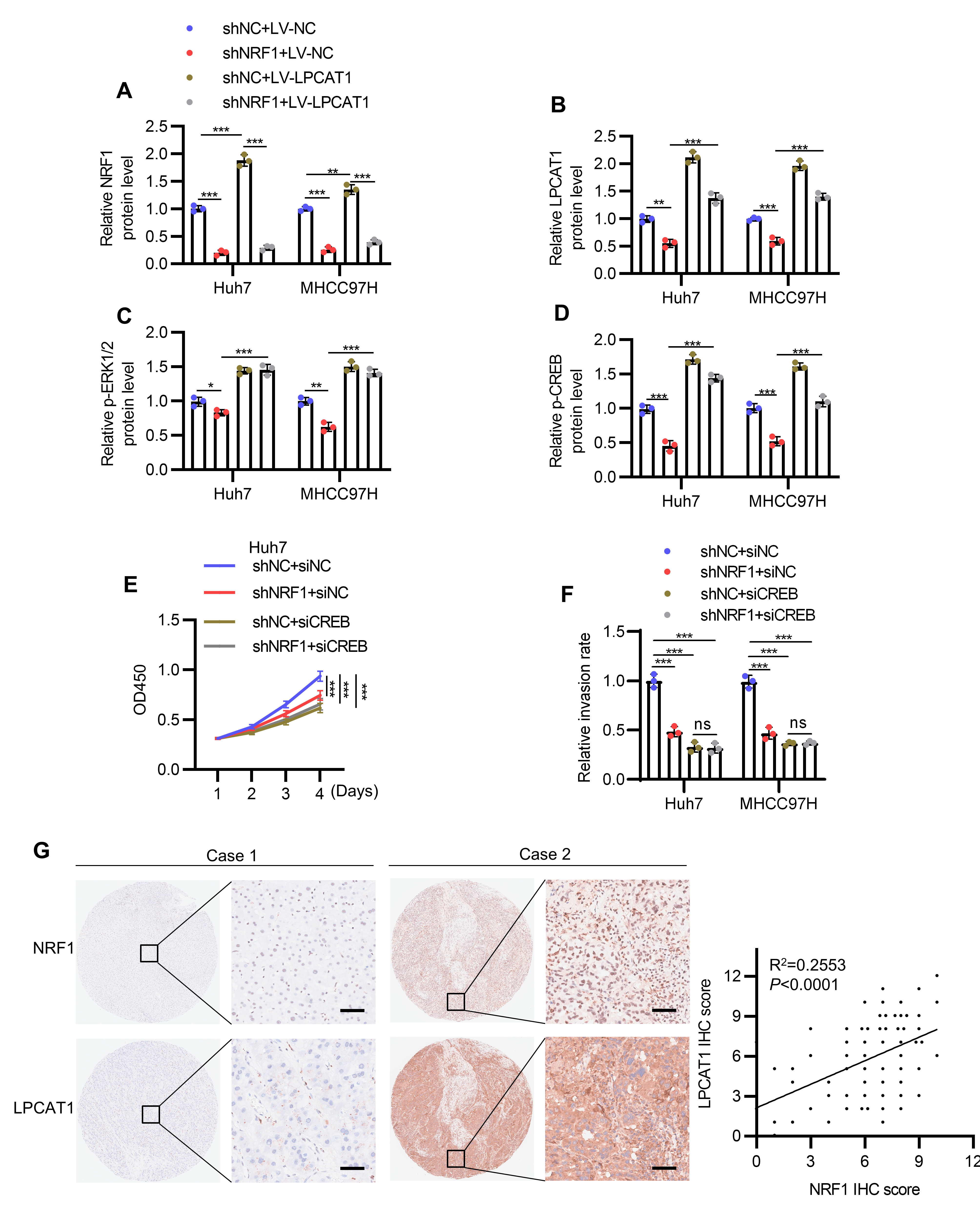

Supplement: Supplementary file 7 — Additional file 7 Figure S7. (A-D) Quantitative results of the protein levels after NRF1 knockdown and LPCAT1 overexpression. Related to Figure 6I. (E-F) The proliferation and invasion ability after NRF1 and CREB knockdown were analyzed by CCK8 (E) and transwell assay (F). (G) Representative IHC results of NRF1 and LPCAT1 and their expression correlation in our HCC tissue microarray. Scale bar: 50 μm. [file 13062_2023_428_MOESM7_ESM.tif]

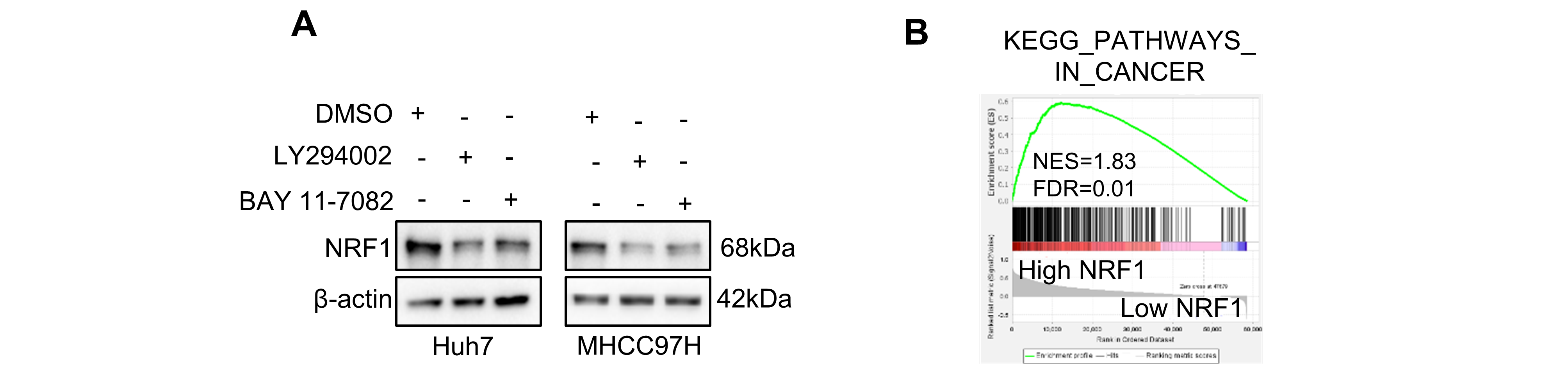

Supplement: Supplementary file 8 — Additional file 8 Figure S8. (A) Western blot result of NRF1 protein level after LY294002 or BAY 11-7082 administration. (B) GSEA with KEGG pathways in cancer gene set between high NRF1 and low NRF1 group in TCGA-LIHC dataset. *p<0.05, **p<0.01, ***p<0.001 [file 13062_2023_428_MOESM8_ESM.tif]
